# Insights into the dynamics of antibiotic resistance genes in the human gut microbiome across populations

**DOI:** 10.1186/s13099-026-00860-2

**Published:** 2026-09-15

**Authors:** Deepika Pateriya, Anjli Tanwar, Vineet K. Sharma

**Affiliations:** MetaBioSys Group, Department of Biological Sciences, Indian Institute of Science Education and Research Bhopal, Bhopal, India

**Keywords:** Antibiotic resistance, Gut microbiome, Oral microbiome, Metagenome, Genome, health, Disease

## Abstract

The human microbiome serves as a reservoir of antibiotic resistance genes (ARGs), collectively known as the resistome, which has crucial implications for human health. However, the distribution of ARGs across diverse bacterial taxa and their variation across populations, disease states, and body sites remain less well understood. Here, we comprehensively profiled the human resistome using genomic and metagenomic data. Our analysis included 4,744 species-representative gut bacterial genomes and 452 oral bacterial genomes, along with gut metagenomic data from 10,230 individuals across 58 studies encompassing 5,388 healthy and 4,842 disease-associated samples, including underexplored non-Western cohorts. Our analysis revealed variation in the gut resistome across population groups and countries. The oral microbiome exhibited a distinct resistome profile with lower ARG prevalence compared to the gut. Across multiple datasets, ARG abundance was generally higher in inflammatory bowel disease samples compared to healthy samples. Pathogenic taxa such as *Enterobacter*, *Citrobacter*, *Escherichia*, and *Klebsiella* carried the highest number of ARGs, including clinically relevant ARGs, whereas abundant commensals like *Bacteroides* and *Prevotella* contributed to the baseline resistome. Notably, population-level differences in ARG composition appeared to be linked to microbial community structure. Shared ARGs between commensal and pathogenic bacteria provided clues to horizontal gene transfer. These findings provide crucial insights into the ecological and population-level factors shaping the gut resistome, highlighting the roles of both pathogens and commensals in the maintenance and dissemination of antimicrobial resistance.

**Supplementary Information:**

The online version contains supplementary material available at https://doi.org/10.1186/s13099-026-00860-2.

## Introduction

Antibiotic resistance is a growing health concern globally, which affects the effectiveness of antibiotic treatment in various diseases, and is estimated to cause 10 million deaths annually by 2050 [1]. While the clinical spotlight traditionally falls on pathogenic bacteria, human commensals have emerged as a significant reservoir of antibiotic resistance genes (ARGs) [2]. These commensals can acquire and disseminate ARGs through horizontal gene transfer (HGT), contributing to resistome expansion and potentially transferring resistance to opportunistic pathogens [2, 3]. Studies have shown that antibiotic resistance is not a modern phenomenon; rather, it predates the clinical use of antibiotics, likely providing a selective advantage and serving as an ancient microbial survival strategy [4, 5]. Factors, including selective pressures from antibiotic exposure, dietary factors, and environmental contamination, have further shaped the distribution and diversity of ARGs in the human gut microbiome [6].

In addition to the gut, the oral microbiome is increasingly recognized as a hotspot for ARGs, with evidence of ARG exchange among oral and gut microbial communities [7, 8]. The oral microbiome, often overlooked in AMR surveillance, is a critical interface between the external environment and the gut, with the potential to harbor and transfer ARGs to the gut and potentially to other pathogens. Understanding how ARGs are distributed across microbial taxa, including commensals and pathogens, and across different populations and disease conditions, is essential for designing effective strategies to overcome the spread of resistance, enhance the efficacy of antibiotics, and promote human health.

In the past decade, numerous studies have explored the gut resistome using large-scale metagenomic data [9–11]. Many of these studies focused on Western populations, overlooking microbiomes from underrepresented non-Western regions like Peru, India, Tanzania, and Madagascar. Moreover, only a few studies have systematically analyzed ARGs in the oral microbiome or investigated population-level variation in individual ARGs across health and disease states [12]. To address these gaps, we leveraged large-scale genomic and shotgun metagenomic datasets to characterize ARGs across global populations. Our dataset included 289,232 metagenome-assembled and isolate-derived gut bacterial genomes from 101 countries, 452 oral-derived bacterial genomes, and 10,230 publicly available gut metagenomes from 58 studies across 35 countries, including 5,388 healthy individuals and 4,842 patients with various diseases. While population-level analyses were performed using gut genomic and metagenomic datasets, the oral microbiome was analyzed using genome catalog to provide complementary insights into ARG distribution. By incorporating datasets from diverse populations and integrating genome- and metagenome-based analyses, our study provides a comprehensive understanding of the global distribution of ARGs, uncovering population-specific and ecological patterns of antimicrobial resistance variation across health and disease conditions.

## Methods

### Retrieval of human gut and oral bacterial genome datasets

To assess the distribution of ARGs within human bacterial genomes, we analyzed publicly available gut and oral bacterial genome data derived from various populations. The gut bacterial genomes were obtained from the Unified Human Gastrointestinal Genome v2.0.2 (UHGG) catalog, consisting of 289,232 metagenome-assembled genomes (MAGs) and isolate genomes corresponding to 4,744 species representatives [13]. Genome sequences of these 4,744 species representatives were retrieved from the MGnify portal (https://www.ebi.ac.uk/metagenomics/genome-catalogues/human-gut-v2-0-2). The protein sequences derived from the UHGG genomes were retrieved from the Unified Human Gastrointestinal Protein (UHGP) catalog, which includes non-redundant protein sequences clustered at 100% identity [13]. The oral genomic data was obtained from the Human oral v1.0.1 catalog from the MGnify portal (https://www.ebi.ac.uk/metagenomics/genome-catalogues/human-oral-v1-0-1). This includes sequences of 452 oral species representative and non-redundant protein sequences (clustered at 100% identity) from 1,225 corresponding MAGs and isolates genomes. Corresponding metadata files that include taxonomy assigned via the genome taxonomy database (GTDB), sample details such as country of origin, continent, accession IDs, and other details were retrieved from the MGnify FTP server.

### Retrieval of human gut metagenomic datasets from various populations

**Fig. 1:**
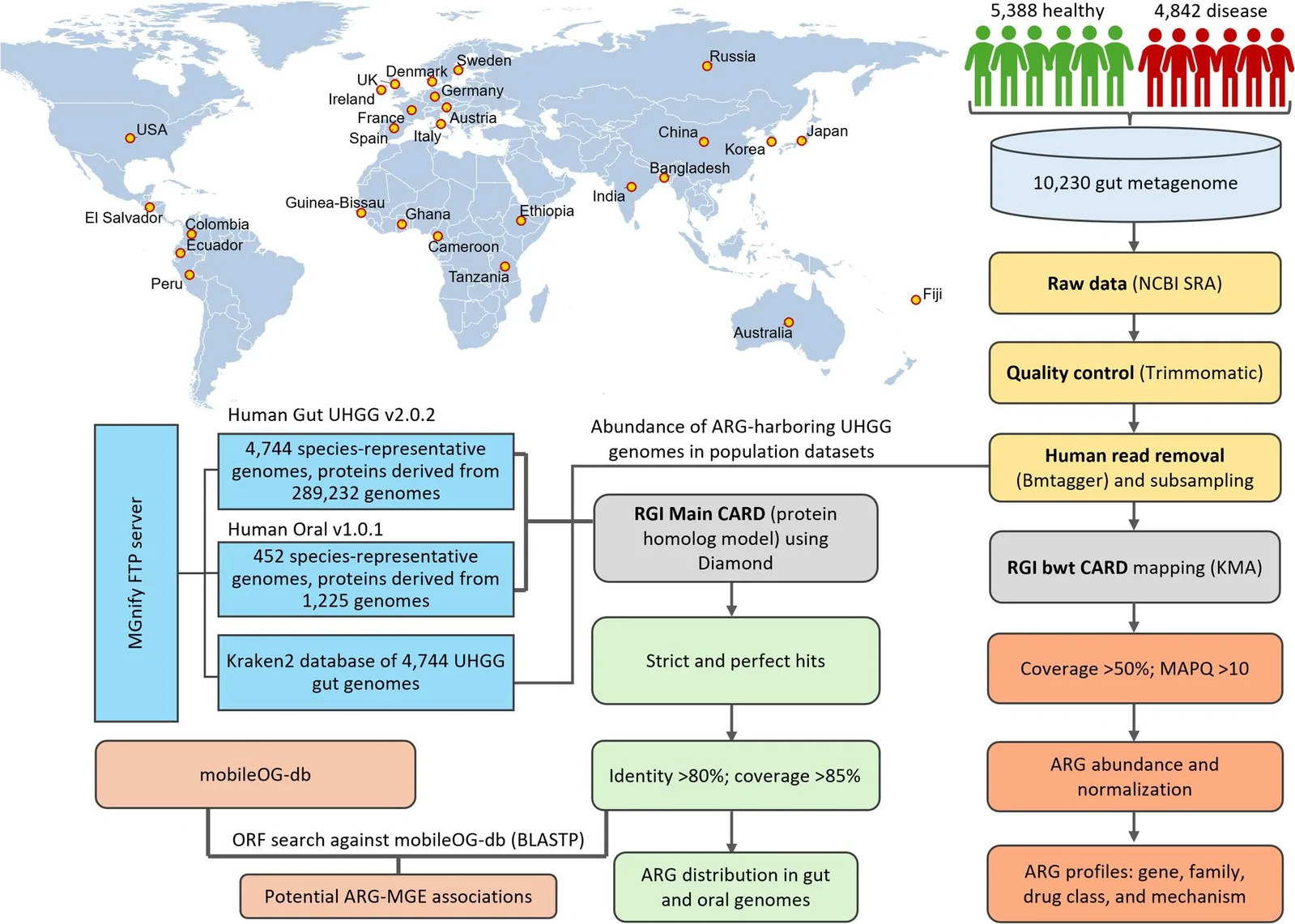
Overview of the study design and analysis pipeline. Steps for processing metagenomic and genomic datasets are shown. The map depicts the geographic distribution of metagenomic samples across 58 studies. Details of the 10,230 metagenomic samples included in the study are provided in Supplementary Table 1

A comprehensive literature review on PubMed was carried out using key terms such as “(gut microbiome OR metagenomics) AND (shotgun)” to retrieve human gut metagenomic shotgun sequencing data. Additionally, repositories such as GMrepo v2 [14] and curatedMetagenomicData [15] were searched for data availability. Studies with publicly available metagenomic data and corresponding metadata were included. For longitudinal studies, baseline samples were selected. The final dataset included 10,230 samples from different populations, including 5,388 healthy samples and 4,842 samples from individuals with disease conditions (Supplementary Table 1, Fig. 1).

### Metagenomic data processing and taxonomy assignment

Raw reads were retrieved from the NCBI Sequence Read Archive (SRA) [16] and the ENA [17]. Reads were quality-checked using FastQC (v0.11.5) [18], and subsequently, uniform preprocessing pipelines were employed to process all datasets. Trimmomatic (v0.36) was used to remove low-quality bases and adapter sequences [19]. The remaining sequences were considered high-quality reads. Potential host genome contamination was removed by aligning high-quality reads against the human reference genome (GRCh38/hg38) using Bmtagger (v3.101) with default parameters [20, 21]. The remaining sequences were considered clean reads.

Subsequently, to standardize sequencing depth across samples, to improve cross-study comparability, and to ensure computational feasibility, clean reads were randomly subsampled using seqkit (v2.2.0) [22], and a maximum of 20 million paired-end reads (20 million read pairs) were selected for further analysis [23]. Since this study integrates datasets generated across multiple independent studies with heterogeneous sequencing depths, subsampling to a common sequencing depth was performed to minimize sequencing-depth-driven variation in ARG detection. The resulting subsampled reads were subjected to taxonomic assignment using Kraken2 (v2.1.0) [24]. A pre-built UHGG database downloaded from the FTP site of MGnify (https://ftp.ebi.ac.uk/pub/databases/metagenomics/mgnify_genomes/human-gut/v2.0.2/) was used as the Kraken2 reference database. Abundance obtained from Kraken2 was subsequently re-estimated using Bracken software [25].

### Antibiotic resistance gene database and genome-based ARG detection

The Comprehensive Antibiotic Resistance Database (CARD, v3.2.8, Release: 2023-10-02) was downloaded [26]. The Resistance Gene Identifier (RGI, v6.0.3) tool was used with the CARD database for ARG annotation. To assess the distribution of ARGs within the human gut and oral bacterial genomes, the RGI *“main”* feature was utilized. This involves the prediction of complete open reading frames (ORFs) using Prodigal (v2.6.3) [27], followed by homolog detection of the predicted protein sequences using the blastp function of DIAMOND (v0.8.36) [28] against reference sequences from the CARD protein homolog model. The ARG prediction employed three criteria: perfect, strict, and loose, based on various types of hits in the homology search. A “perfect” match indicates 100% identity to the reference sequence along its entire length, while a “strict” prediction surpasses the bit-score threshold set by the curated BLASTP bit-score cut-off. “Loose” matches encompass sequences with bit-score below the curated BLASTP bit-score. We considered the hits with “strict” and “perfect” criteria for detecting ARGs in genomes because loose hits may or may not confer AMR. Hits with > 80% identity and > 85% coverage were further selected and used for downstream analysis based on the recommended parameters in previous studies [29].

### ARG detection in metagenomic datasets using read-based annotation

To identify ARGs in metagenomic datasets, reads were mapped against the ARG sequences in the CARD database using the “bwt” function of the RGI tool with default parameters. RGI bwt mapped reads to reference sequences from the CARD protein homolog model using the KMA aligner (v1.4.9) [30]. The resulting gene mapping file was used to filter aligned reads for each sample based on coverage and average mapping quality (MAPQ) score. Aligned reads with at least 50% coverage and a MAPQ score greater than 10 were retained for downstream analysis [31, 32]. Subsequently, ARG read counts were normalized to reads per kilobase per million mapped reads (RPKM) to account for gene length and sequencing depth, using the formula: RPKM = (number of reads mapped to an ARG × 10⁹) / (gene length in base pairs × total number of mapped reads in the sample).

### Mobile genetic elements annotation using MobileOG-db database

To identify mobile genetic elements (MGEs) associated with ARGs, the mobile orthologous group database (mobileOG-db) was used [33]. First, we retrieved all ORFs predicted from ARG-harboring genomes using the RGI prodigal module. These predicted ORF sequences were then used as queries against the MobileOG-db database using the blastp function of DIAMOND software. MGE hits were considered at a minimum sequence identity of 80% and subject coverage of 85% [34]. The identified MGEs were then classified into different categories using the mobileOGs-pl-kyanite.py script. To assess the potential association between ARGs and MGEs, ARG and MGE coordinates were compared within each contig. ARGs located within 100 kilobases (kb) of an MGE, based on the distance between their genomic coordinates, were considered potentially associated, indicating possible co-localization [35].

### Statistical analysis

To evaluate the distribution of ARGs in the human gut microbiome across populations, we compared ARG abundance between healthy individuals from different countries and between disease and control groups within individual case-control studies. Healthy populations were further categorized into groups based on dietary habits, industrialization, and geographic region, following previous microbiome studies [36, 37]. These included: (I) Western industrialized populations (Austria, Denmark, France, Germany, Ireland, Italy, Spain, Sweden, UK, USA); (II) traditional non-Western populations (India, Cameroon, Guinea-Bissau, Fiji, El Salvador, Peru, Ethiopia, Ghana, Tanzania); and (III) urban poor communities (Peruvian slum). Additionally, countries, including China, Colombia, Japan, South Korea, and Russia, were analyzed as independent groups, as these could not be easily categorized within the broader groups. Western industrialized and traditional non-Western populations are hereafter referred to as Western (W) and non-Western (NW), respectively.

RPKM abundance of ARGs in metagenomic samples was converted to relative abundance by dividing the abundance of each ARG by the total ARG abundance within each sample. After adding a pseudocount of 1 to prevent non-finite values from log_2_(0), relative abundance was log-transformed as log_2_(relative abundance + 1).

Bray-Curtis distance matrix was calculated using log-transformed relative abundance data. Principal coordinates analysis (PCoA) was performed based on Bray-Curtis distances in R (v4.3.1). Differences in ARG composition between groups were assessed using PERMANOVA implemented through the adonis2 function in the vegan R package (999 permutations). To account for study-level heterogeneity, permutations were constrained within Study ID strata (strata = Study ID). To evaluate the robustness of normalization approaches, PCoA analysis was also performed using RPKM-based abundance values without conversion to relative abundance. A two-sided Mantel test (Spearman correlation, 999 permutations) was performed to assess the similarity between Bray-Curtis distance matrices derived from RPKM based and log-transformed relative abundance data using the qiime diversity mantel command in QIIME2 (v2023.7.0) [38].

Differential abundance analysis was performed using MaAsLin2, with study ID included as a fixed effect to control for study-specific variation. LEfSe was used to identify differentially abundant ARGs between various case-control groups [39]. ARG abundance distributions (log_2_(relative abundance + 1)) were visualized using boxplots generated with the ggplot2 package in R (v4.3.2) using RStudio. Pairwise differences between groups were assessed using the two-sided Wilcoxon rank-sum test, and p-values were adjusted for multiple comparisons using the Benjamini-Hochberg method. For higher-level analyses, including ARG family, drug class, and resistance mechanism profiling, abundance values were calculated by summing the RPKM abundances of ARGs belonging to the respective categories.

## Results

### Taxonomic distribution of ARG-rich gut bacterial genomes highlights the dominance of opportunistic pathogens

To analyze the distribution of ARGs in the human gut bacteria, a total of 4,744 gut bacterial species representatives from the MGnify UHGG catalog were examined for the presence of ARG homologs using the RGI tool with the CARD database. Additionally, protein catalog from 289,232 genomes corresponding to these species representatives were analyzed to assess the distribution of ARG homologs across genomes. Our results showed that 857 genomes out of 4,744 harbored homologs for at least one CARD-annotated ARG (Supplementary Table 2).

The ARG prevalence at the phylum level revealed that Proteobacteria contain the highest proportion, with 181 out of 353 species (51%) with ARGs. This was followed by Firmicutes (150/529 species, ~ 28%), Firmicutes A (311/1906 species, ~ 16%), and Bacteroidota (96/619 species, ~ 15%) (Supplementary Table 2).

**Fig. 2:**
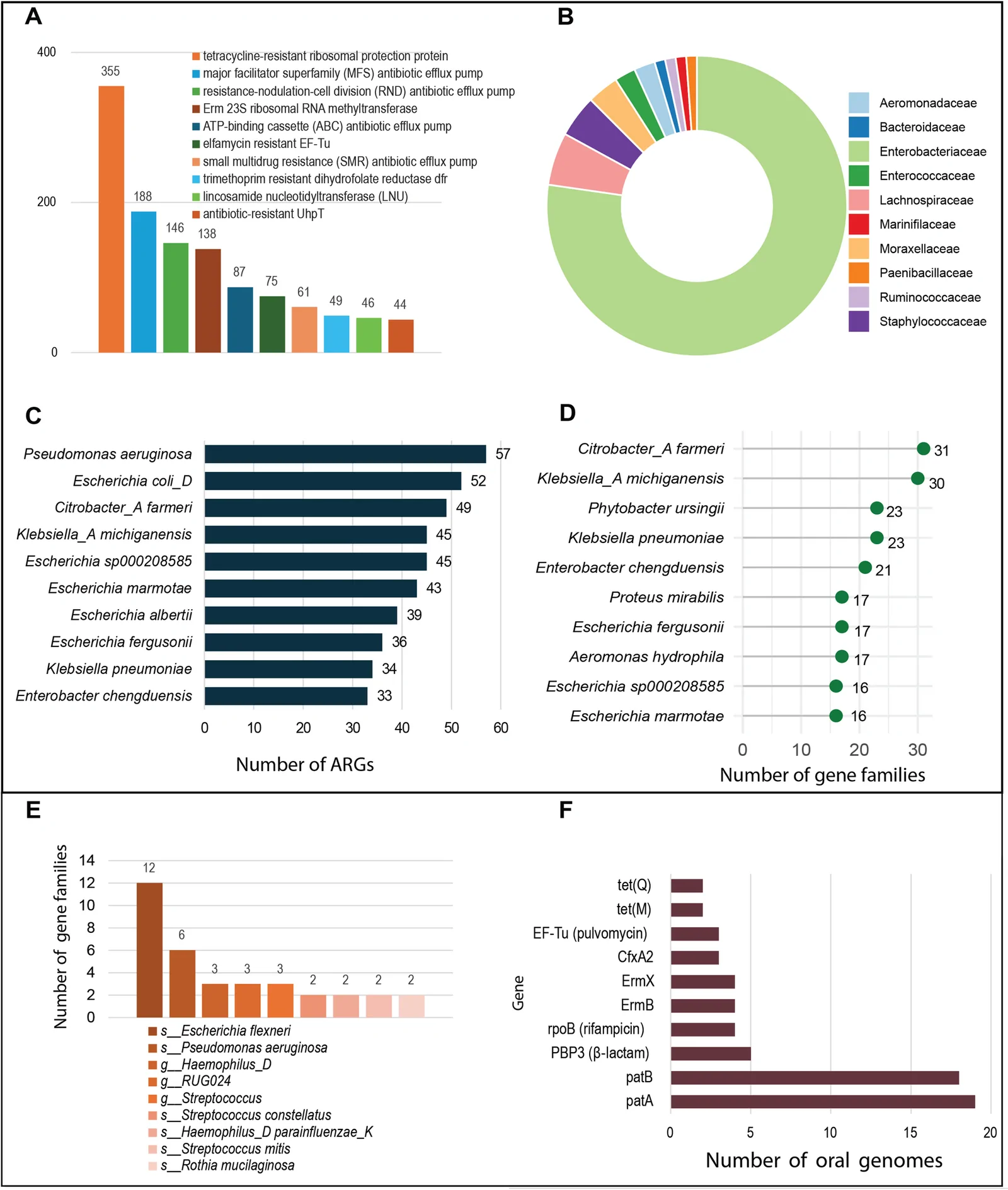
Prevalence and distribution of antibiotic resistance genes (ARGs) among gut and oral bacterial genomes. **(A)** Distribution of major antimicrobial resistance (AMR) families across 857 gut species representative genomes obtained from the UHGG catalog. **(B)** Family-level distribution of the top 100 gut genomes harboring the highest number of ARGs. **(C)** Top 10 gut bacterial species with the highest total number of ARGs. **(D)** Top 10 gut species with the highest number of AMR families. **(E)** Top 9 oral species representative genomes carrying the highest number of ARG families. **(F)** Prevalence of specific ARGs across oral bacterial genomes

Among the 857 gut species representatives, the most prevalent AMR families included tetracycline-resistant ribosomal protection proteins (355 species) and Erm 23 S ribosomal RNA methyltransferases (138 species). Efflux pump families were also widespread, with major facilitator superfamily (MFS) antibiotic efflux pumps identified in 188 species, resistance-nodulation-cell division (RND) antibiotic efflux pumps in 146 species, and ATP-binding cassette (ABC) antibiotic efflux pumps in 87 species. Other AMR families showed low prevalence and were found in species ranging from one to 75 (Fig. 2A, Supplementary Table 2).

Although efflux pumps are commonly annotated across bacterial genomes and often play a role in intrinsic physiological functions, their overexpression can contribute to reduced antibiotic susceptibility. We therefore retained them in our analysis to provide a complete resistome profile, while interpreting their presence carefully.

At the family level, the highest number of species with ARGs belonged to Lachnospiraceae (143 species), Enterobacteriaceae (114 species), and Bacteroidaceae (66 species) (Supplementary Table 2). The tetracycline-resistant ribosomal protection protein family was most prevalent among the members of Lachnospiraceae and Bacteroidaceae, and was identified in 105 and 39 species, respectively. In Enterobacteriaceae, a broad spectrum of clinically relevant ARG families were detected, including pmr phosphoethanolamine transferase (colistin resistance), qnr quinolone resistance proteins, fluoroquinolone-resistant gyrB, fosfomycin-inactivating enzymes, and ACT β-lactamases. Although RND, MFS, and ABC efflux pumps were also common in Enterobacteriaceae, these might reflect intrinsic physiological roles (Supplementary Table 2).

Analysis of the top 100 genomes harboring the highest number of ARGs revealed that 68 species belonged to the Enterobacteriaceae family (Supplementary Table 2, Fig. 2B). Among these, the top 10 genomes with the highest number of ARGs include gram-negative bacteria, such as *Pseudomonas aeruginosa*, *Citrobacter_A farmeri*, *Klebsiella pneumoniae*, *Klebsiella_A michiganensis*, *Enterobacter chengduensis*, and five species of the *Escherichia* genus (Fig. 2C). These taxa harbored ARGs for more than 20 drug classes. Notably, most of these species are opportunistic pathogens linked to hospital-acquired infections and antimicrobial resistance. Furthermore, among 857 genomes, the top 10 species with the highest number of AMR families are shown in Fig. 2D. A detailed summary of the presence and prevalence of different ARGs and AMR gene families across gut bacterial genomes is provided in Supplementary Table 3.

We further examined the distribution of clinically relevant ARGs across genomes. Vancomycin resistance genes (*vanR*,* vanH*,* vanX*,* vanXY*,* vanW*,* vanU*, and *vanY*) were restricted to the phylum Firmicutes, primarily among Clostridia and Bacilli classes. These genes were present in genera such as *Enterococcus*, *Blautia*, *Coprococcus*, *Ruminococcus*, *Massilimaliae*, and *Neobacillus*. Clinically important β-lactamases such as *CTX-M* were found in *Kluyvera ascorbata* and *Enterobacter chengduensis*. Carbapenemases, including *KPC* and *NDM-1*, were confined to members of the Enterobacterales order, whereas *cfiA4* and *cepA* were detected in *Bacteroides fragilis*. Additionally, colistin resistance determinants (*alm*, *ArnT*, and *MCR*), as well as *AAC(6’)-Ib* and *QnrS*, which confer resistance to aminoglycosides and quinolones, respectively, were limited to Gammaproteobacteria (Supplementary Table 3). Overall, these clinically important ARGs were predominantly associated with pathogenic taxa with limited distribution among commensal gut bacteria.

### ARG distribution in oral bacterial genomes shows lower prevalence and distinct taxonomic patterns

To understand the ARG distribution in human oral bacteria, 452 oral species representative genomes and ORFs predicted from 1,225 corresponding genomes obtained from the MGnify oral genome catalog were examined. Among the 452 genomes, 64 genomes harbored homologs for at least one ARG (Supplementary Table 4). Among these, *Escherichia flexneri* and *Pseudomonas aeruginosa* carried the highest number of ARG families (Fig. 2E).

The identified ARG homologs in *E. flexneri* were from various families such as AmpC-type beta-lactamase (cephalosporin, penam), undecaprenyl pyrophosphate-related proteins (peptide antibiotic), Pmr phosphoethanolamine transferase (peptide antibiotic) and multiple intrinsic efflux pump families (MFS, SMR, RND, and ABC). Similarly, *P. aeruginosa* harbored homologs for OXA beta-lactamase (carbapenem, cephalosporin), chloramphenicol acetyltransferase (phenicol antibiotic), Pmr phosphoethanolamine transferase and multiple efflux pumps (Supplementary Table 4).

Among the most prevalent ARG homologs, *patA*, *patB*, *PBP3*, *rpoB*, *ErmB*, *ErmX*, and *CfxA2* were detected in the highest numbers of oral genomes (Fig. 2F). *patA* and *patB*, which are associated with fluoroquinolone resistance, were found to be restricted to the members of the *Streptococcus* genus. The presence of efflux pump genes may reflect their intrinsic physiological roles in the oral microbiome.

The *CfxA2* gene, which confers resistance to cephamycin, was found in the *Prevotella* species, including *Alloprevotella tannerae*, *Prevotella jejuni*, and *Prevotella histicola*. *PBP3* homologs were identified in the *Haemophilus* genus, whereas *rpoB*, which confers resistance to rifamycin, was detected in *Bifidobacterium* and *Scardovia wiggsiae* (Supplementary Table 4).

As suggested in previous research, oral microbes may acquire ARGs through frequent exposure to locally administered antibiotics, such as those used for treating periodontal diseases [8]. For example, several ARGs identified in oral genomes correspond to antibiotics commonly used in dental practice, including β-lactams (amoxicillin, cephalosporins), metronidazole, clindamycin, macrolides, and tetracyclines. These antibiotics are routinely prescribed for oral infections. In contrast, gut bacteria are more likely to acquire ARGs through HGT within the dense microbial community and through continuous exposure to antibiotics via diet [40, 41].

Overall, the oral microbiome showed a distinct ARG profile, with fewer ARG types detected than in the gut microbiome. However, this observation is based on analysis of two separate genome catalogs and differences in catalog size, genome representation, sequencing depth, and assembly strategies between the UHGG (gut) and oral genome catalogs may influence the apparent ARG profiles.

### ARGs associated with tetracycline resistance were prevalent in gut microbiome across populations

To understand the distribution of ARGs in the human gut microbiome across the global population, 10,230 publicly available gut metagenomic samples (5,388 healthy and 4,842 with various diseases) from 58 studies across 35 countries were analyzed. A total of 1,129 unique ARGs (136 AMR families, 115 drug classes, and 10 resistance mechanisms) were detected in at least one of the 5,388 healthy samples. The top five abundant ARGs that were ubiquitous across different populations and disease conditions included *Bifidobacterium adolescentis rpoB* mutants, which confer resistance to rifamycin, along with tetracycline resistance genes, i.e., *tet(40)*, *tet(Q)*, *tet(O)*, and *tet(W)*. These ARGs were widely distributed across populations and health conditions (Table 1, Supplementary Table 5).

**Table 1:** The top five most prevalent antibiotic resistance genes (ARGs) across populations, with their corresponding AMR families, drug classes, and resistance mechanisms

ARG	AMR Gene Family	Drug Class	Resistance Mechanism	Number of samples	Percentage of samples
*Bifidobacterium adolescentis* *rpoB*	rifamycin-resistant beta-subunit of RNA polymerase (rpoB)	rifamycin antibiotic	antibiotic target alteration, antibiotic target replacement	9279	90.70%
*tet(40)*	MFS antibiotic efflux pump	tetracycline antibiotic	antibiotic efflux	9554	93.39%
*tet(Q)*	tetracycline-resistant ribosomal protection protein	tetracycline antibiotic	antibiotic target protection	9798	95.78%
*tet(O)*	tetracycline-resistant ribosomal protection protein	tetracycline antibiotic	antibiotic target protection	9904	96.81%
*tet(W)*	tetracycline-resistant ribosomal protection protein	tetracycline antibiotic	antibiotic target protection	10101	98.74%

### Distinct clustering of ARGs among healthy populations reflects variation in the global gut resistome profile

To investigate the distribution of ARGs in the gut microbiome of healthy individuals, we analyzed ARG profile across populations, broadly grouped into Western (W) populations (Austria, Denmark, France, Germany, Ireland, Italy, Spain, Sweden, UK, USA), non-Western (NW) populations (India, Cameroon, Guinea-Bissau, Fiji, El Salvador, Peru, Ethiopia, Ghana, Tanzania), and urban poor communities (Peruvian slum). In addition, countries, including China, Colombia, Japan, South Korea, and Russia, were analyzed separately, as they could not be clearly assigned to these broader categories. Analyses were performed at both population groups and country levels.

**Fig. 3:**
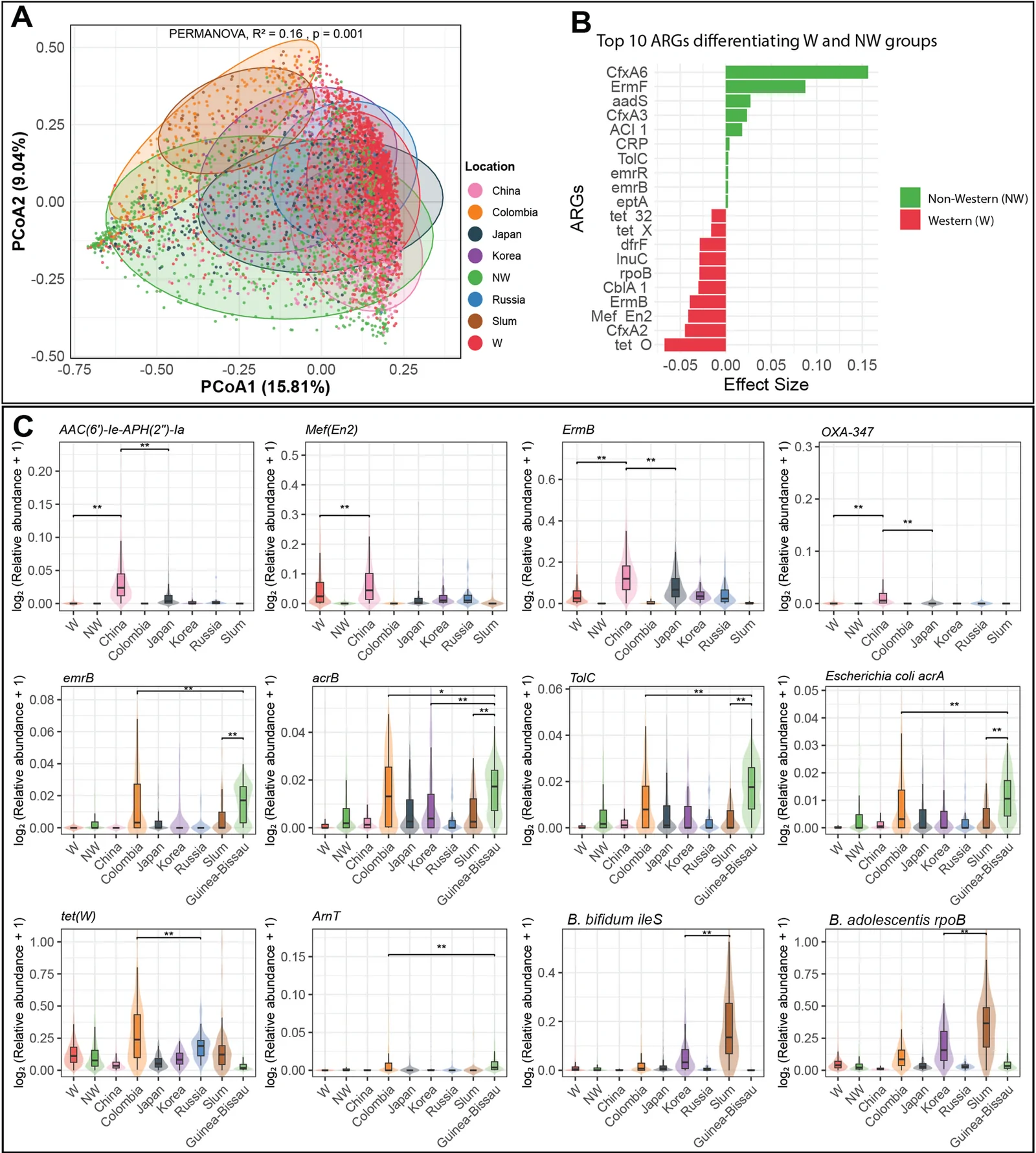
Distribution of ARGs across global populations. **(A)** Principal coordinates analysis (PCoA) based on inter-sample Bray-Curtis distances calculated using log₂ (relative abundance + 1) transformed data showing the distribution of ARGs across China, Colombia, Japan, Korea, Non-Western (NW), Russia, Slum, and Western (W) groups. Statistical significance was assessed using PERMANOVA with stratified permutations (R² = 0.16, *p* = 0.001). **(B)** Top 10 differentially abundant ARGs distinguishing Western and non-Western groups identified using MaAsLin2 analysis. Positive effect sizes indicate enrichment in non-Western populations, whereas negative effect sizes indicate enrichment in Western populations. **(C)** Boxplots showing the distribution of ARGs (log₂ (relative abundance + 1)) across different populations. Pairwise statistical significance between groups was assessed using the two-sided Wilcoxon rank-sum test with Benjamini-Hochberg correction. Asterisks indicate adjusted p-values (*p < 0.05; **p < 0.01). Detailed adjusted *p*-values are provided in Supplementary Table 6

PCoA based on Bray-Curtis distance (using log-transformed relative abundance) showed distinct clustering of healthy samples across population groups (PERMANOVA, R2 = 0.16, p = 0.001) (Fig. 3A). Similar clustering patterns were observed when using RPKM-based abundance values (Supplementary Fig. 1). Mantel test results further indicated a strong correlation between distance matrices derived from RPKM-based abundance and log-transformed relative abundance data (ρ = 0.92, p-value = 0.001), suggesting that overall patterns were preserved (Supplementary Fig. 1). Pairwise PERMANOVA comparisons further supported significant differences in ARG composition between multiple population groups (Supplementary Table 5). Significant differences were observed between non-Western and Western populations (R² = 0.09, p = 0.001), indicating variation in ARG profiles across these groups. Additional significant differences were also observed for several other pairwise comparisons, including Peruvian slum vs Russia, Russia vs Colombia, China vs Colombia, Japan vs Colombia, and Peruvian slum vs Korea (Supplementary Table 5).

Differential abundance analysis using MaAsLin2, with study ID included as a fixed effect to account for study-level variation, revealed significant differences in the relative abundance of ARGs across population groups. When comparing Western vs. non-Western groups, several clinically relevant ARGs showed significantly higher relative abundance in Western populations (FDR-adjusted q < 0.05). These included macrolide-lincosamide-streptogramin (MLS) resistance genes (*ErmB*,* lnuC*), β-lactam resistance genes (*CblA-1*,* CfxA2*), and macrolide resistance gene *Mef(En2)*. In addition, multiple tetracycline resistance genes, including *tet(O)*, *tet(W)* variants, and *tet(X)*, as well as aminoglycoside resistance determinants (*APH(3’)-IIIa*,* aad(6)*) and nucleotidyltransferases (*SAT-4*), also showed higher relative abundance in Western populations. (Fig. 3B, Supplementary Fig. 2, Supplementary Table 6).

In contrast, ARGs such as *CfxA3*, *CfxA6* (β-lactamases), and *ErmF* (MLS resistance) showed higher relative abundance in the non-Western compared to the Western group. (Fig. 3B, Supplementary Table 6). Chinese group exhibited higher relative abundance of aminoglycoside resistance genes (*AAC(6’)-Ie-APH(2’’)-Ia*,* APH(2’’)-If*), and MLS resistance genes (*ErmB*, and *ErmF*,* Erm(52)*) compared to other groups. *OXA-347*, a beta-lactamase of lesser-known clinical significance, and *Mef(En2)* also showed higher relative abundance in the Chinese group (Fig. 3C, Supplementary Fig. 3, Supplementary Table 6).

Colombia showed higher relative abundance of multiple multidrug resistance-associated genes (Fig. 3C, Supplementary Table 6). Notably, components of major efflux systems, including *acrB*, *AcrE*, *AcrF*, *TolC*, and several mdt family genes (*mdtB*, *mdtE*), along with *emrA* and *emrB*, showed higher relative abundance. In addition, regulatory genes such as *marA* and *ramA*, which are known to modulate efflux activity, also showed higher relative abundance. Beyond efflux systems, several clinically relevant ARGs exhibited higher relative abundance, including β-lactam resistance genes such as *Escherichia coli ampC* and macrolide resistance determinants *mef(H)*. Tetracycline resistance genes, including *tet(W)* and *tet(A)*, also showed higher relative abundance in this population. Genes associated with membrane modification and reduced permeability, such as *ArnT*, *eptA*, and *LptD*, also showed higher relative abundance in this population. Together, these patterns suggest a higher representation of multidrug resistance-associated genes in the Colombian dataset.

Country-level analysis revealed that samples from Guinea-Bissau showed higher relative abundance of efflux and membrane-associated resistance determinants, including *acrB*, *TolC*, *emrA*, *emrB*, mdt family genes, *msbA*, *gadX*, *leuO*, and *eptA*, along with pathogen-associated genes such as multiple *Klebsiella pneumoniae* genes (*OmpK37*, *KpnG*,* KpnE*, and *KpnF*) (Supplementary Table 6).

Although many of these genes are part of the intrinsic efflux system rather than acquired resistance genes, their enrichment may indicate differences in microbial communities and population-level factors. These patterns are consistent with previous reports indicating that Guinea-Bissau lacks a well-implemented National AMR Action Plan [42, 43], and inadequate sanitation, limited access to healthcare, and unregulated antibiotic use may contribute to ARG dissemination in this population [44]. Furthermore, a higher relative abundance of *Bifidobacterium ileS* (mupirocin resistance) and *rpoB* mutants was observed in the Peruvian slum population compared to other groups (Fig. 3C).

### Distribution of AMR families across populations

**Fig. 4:**
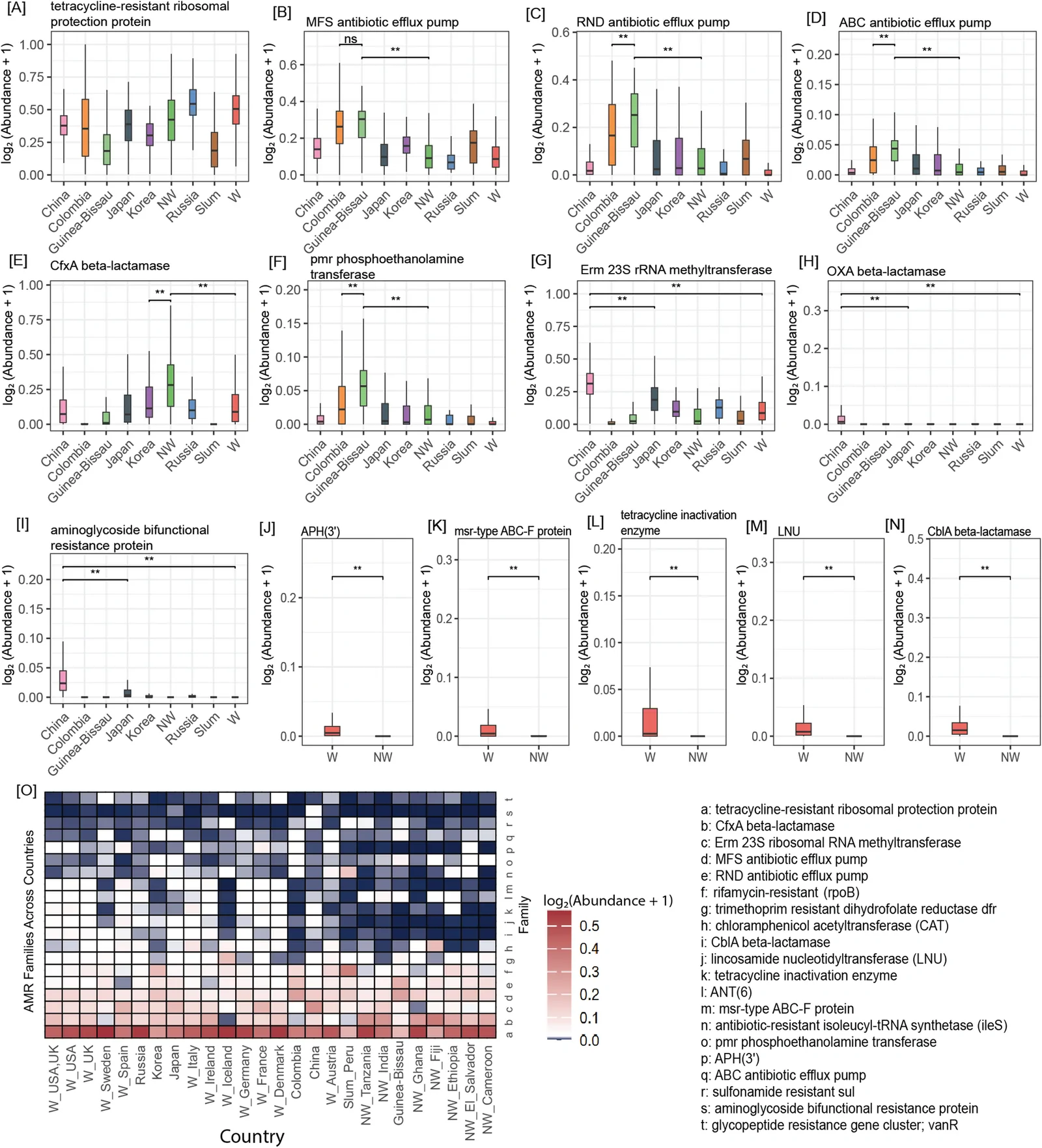
Distribution of antimicrobial resistance (AMR) families across population groups. **(A-N)** Boxplots showing the abundance (log₂(abundance + 1)) of selected AMR families across different populations. Pairwise statistical significance between groups was assessed using the two-sided Wilcoxon rank-sum test. Asterisks indicate p-values (**p < 0.01), while “ns” indicates non-significant differences. The box bounds and central line represent the interquartile range (25th-75th percentiles) and median, respectively. **(O)** Heatmap showing the distribution of the top abundant AMR families across countries belonging to population groups based on summed RPKM abundance values. Color intensity represents log₂(abundance + 1). Detailed adjusted p-values are provided in Supplementary Table 6

To understand broader AMR patterns, ARGs were grouped into AMR families. AMR families such as tetracycline-resistant ribosomal protection proteins and efflux pump families, including MFS, RND, and ABC efflux pumps, showed prevalence across population groups (Fig. 4A, B, C, and D).

Several clinically relevant AMR families showed distinct distribution patterns across populations. In the non-Western group, CfxA β-lactamase showed significantly higher abundance compared to other groups (Fig. 4E). Efflux pump families (RND, MFS, and ABC) were enriched in Colombia and Guinea-Bissau datasets, consistent with gene-level observations (Fig. 4B and C). In addition, membrane modification-associated families, including phosphoethanolamine transferases (e.g., EptA/PmrC), showed higher abundance in Colombia and Guinea-Bissau (Fig. 4F). These genes have previously been associated with reduced susceptibility to polymyxin antibiotics.

Erm 23 S rRNA methyltransferase, a major contributor to MLS resistance, was prevalent across groups but showed higher abundance in the Chinese group (Figure 4G). Members of the Erm methyltransferase family modify 23 S rRNA of bacterial ribosomes, conferring cross-resistance to macrolides and multiple unrelated antibiotics. Previous studies have reported ErmB-mediated resistance as a major contributor to macrolide resistance in European countries [45], with macrolide resistance being reportedly higher in China than in other Western countries [46]. Chinese groups also showed enrichment of additional clinically relevant AMR families. These include OXA β-lactamase (Figure 4H, Supplementary Figure 3C), a Class D β-lactamase, which confers resistance to cefotaxime, imipenem, and penicillin, and has been detected in the human gut microbiome, wild and domestic animals, and wastewater [47, 48]. The aminoglycoside bifunctional resistance protein family was also enriched in Chinese datasets (Figure 4I). This might be attributed to the widespread use of aminoglycosides in poultry and swine farming, which promotes the selection and persistence of these resistance determinants [49].

Further, the Western group showed higher abundance of AMR families such as APH(3’) (aminoglycoside resistance), MSR-type ABC-F proteins (macrolide resistance), tetracycline inactivation enzymes, lincosamide nucleotidyltransferase (LNU), and CblA β-lactamase compared to the non-Western group (Fig. 4J and N). A heatmap visualisation of the top 20 most represented AMR families across countries is shown in Fig. 4O.

### Global variation in AMR drug classes and resistance mechanisms among healthy individuals

**Fig. 5:**
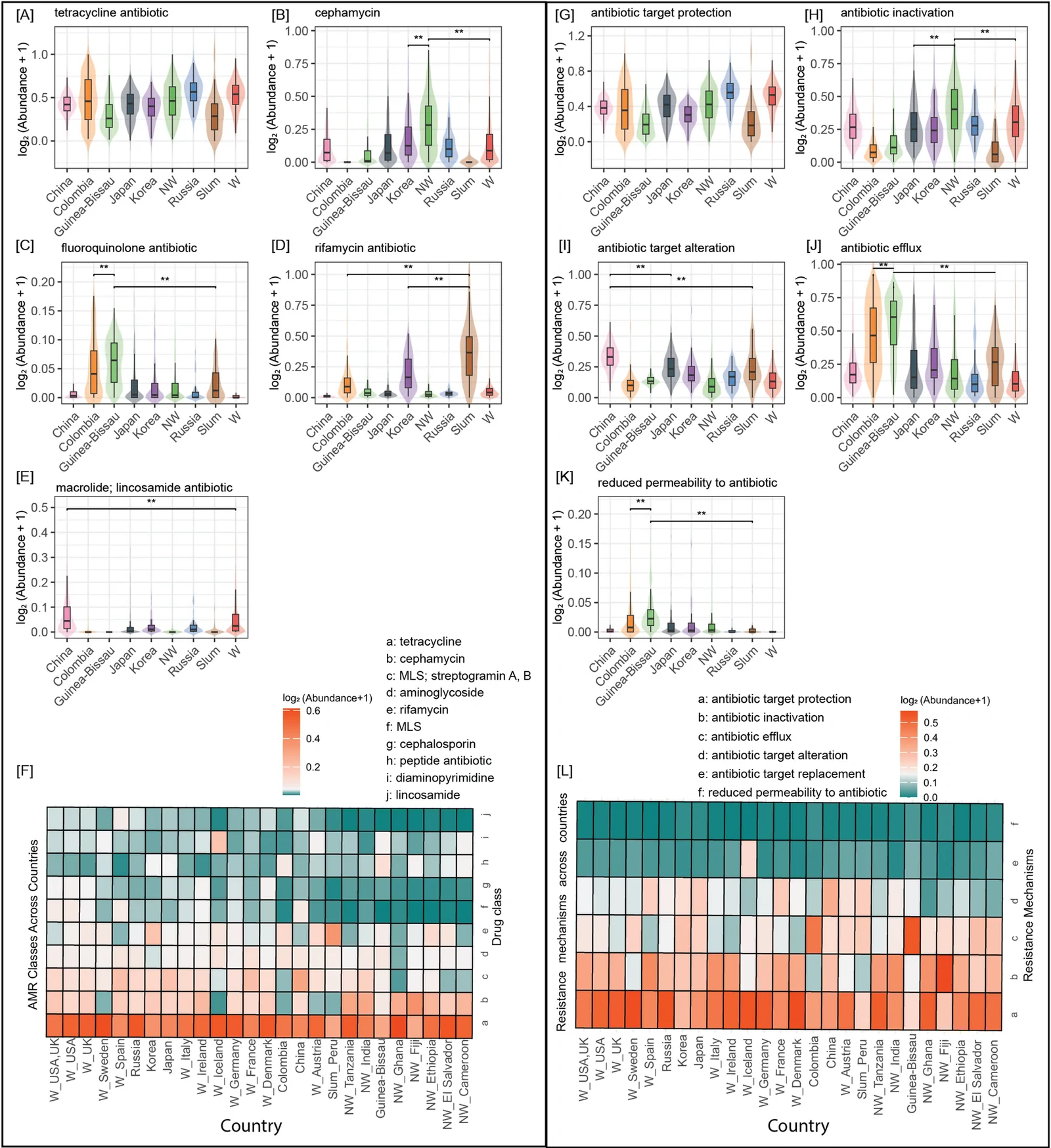
Distribution of antimicrobial resistance drug classes and resistance mechanisms across population groups. (**A-E**) Boxplots showing the abundance (log₂ (abundance + 1)) of selected antimicrobial resistance drug classes across different populations. (**F**) Heatmap showing the distribution of the most abundant antimicrobial resistance drug classes across countries corresponding to population groups based on summed RPKM abundance values. Color intensity represents log₂(abundance + 1). (**G**-**K**) Boxplots showing the abundance (log₂ + 1) of selected antimicrobial resistance mechanisms across different populations. (**L**) Heatmap showing the distribution of the most abundant resistance mechanisms across countries and population groups based on summed RPKM abundance values. Color intensity represents log₂(abundance + 1). Pairwise statistical significance between groups was assessed using the two-sided Wilcoxon rank-sum test. Asterisks indicate p-values (**p < 0.01). Detailed adjusted p-values are provided in Supplementary Table 6

To evaluate resistance patterns across antibiotic classes, ARGs were grouped by their respective drug classes. Resistance to tetracyclines showed widespread representation across population groups, suggesting that tetracycline resistance determinants are commonly present in the gut resistome (Fig. 5A). Distinct group-specific patterns were observed across multiple drug classes. The non-Western group showed a comparatively higher abundance of cephamycin resistance than the Western population group, although this class was detected across all groups (Fig. 5B). Fluoroquinolone resistance showed higher abundance in Guinea-Bissau and Colombian datasets (Fig. 5C), whereas rifamycin resistance showed markedly higher abundance in Peruvian slum populations compared to other groups (Fig. 5D). Resistance to MLS antibiotics was higher in Western and Chinese groups compared to other groups. (Fig. 5E). A heatmap visualisation of the top 10 most represented drug classes across countries is shown in Fig. 5F.

To further understand the functional strategies underlying the observed AMR patterns, we examined ARGs at the level of resistance mechanisms. Antibiotic target protection was represented across all population groups, consistent with the widespread distribution of tetracycline resistance genes (Fig. 5G). Distinct population-level differences were observed for other resistance mechanisms. For example, antibiotic inactivation showed a significantly higher representation in the non-Western compared to the Western group (Fig. 5H), and antibiotic target alteration showed a higher abundance in the Chinese group (Fig. 5I). Efflux-mediated resistance exhibited markedly higher representation in Guinea-Bissau and Colombian datasets, with additional enrichment in Peruvian slum populations, consistent with the higher relative abundance of multidrug resistance-associated efflux systems observed at the gene and family levels (Fig. 5J). Reduced permeability mechanisms showed comparatively higher abundance in Guinea-Bissau and Colombian datasets (Fig. 5K). These mechanisms have previously been linked to decreased antibiotic uptake in bacteria. A heatmap visualisation of the top 6 most represented resistance mechanisms across countries is shown in Fig. 5L.

### Comparison of gut resistome profiles between younger and older age groups

Previous studies have suggested that the gut microbiome of children harbors higher relative abundance of ARGs compared to older age groups, likely due to differences in microbiome composition and antibiotic exposure during early life. To examine this aspect, we compared gut resistome profiles between children (age < 4 years) and individuals older than 4 years across studies that included both age groups.

Within individual studies, children generally showed slightly higher mean ARG relative abundance compared to older individuals, however, these differences were not significant in most cases (Table 2). However, an exception was observed in the DhakanDB-2019 study, where children had a 3.5-fold higher ARG abundance (mean: 5583.21) compared to individuals > 4 years (mean: 1581.52). However, this study included only five children under the age of four, which may limit the generalizability of this observation.

**Table 2:** Mean ARG abundance of children (age < 4 years) and individuals aged > 4 years across multiple studies

Study ID	Disease condition	Group	Mean ARG Abundance (age < 4 years)	Mean ARG Abundance (age > 4 years)	Reference
DhakanDB-2019	Healthy	NW	5583.21	1581.52	[50]
TettA-2019	Healthy	NW	1661.98	1394.89	[51]
VishnuP-2019	Healthy	NW	1421.71	1734.21	[37]
PehrssonE-2016	Healthy	Slum	1496.18	1114.83	[52]
PehrssonE-2016	Healthy	NW	1220.93	1202.65	[52]
DanZ-2020	Healthy	W	2216.02	2470.43	[53]
WanY-2022	Healthy	W	1274.16	1400.51	[54]

### Increased ARG abundance in IBD patients compared to healthy individuals

**Fig. 6:**
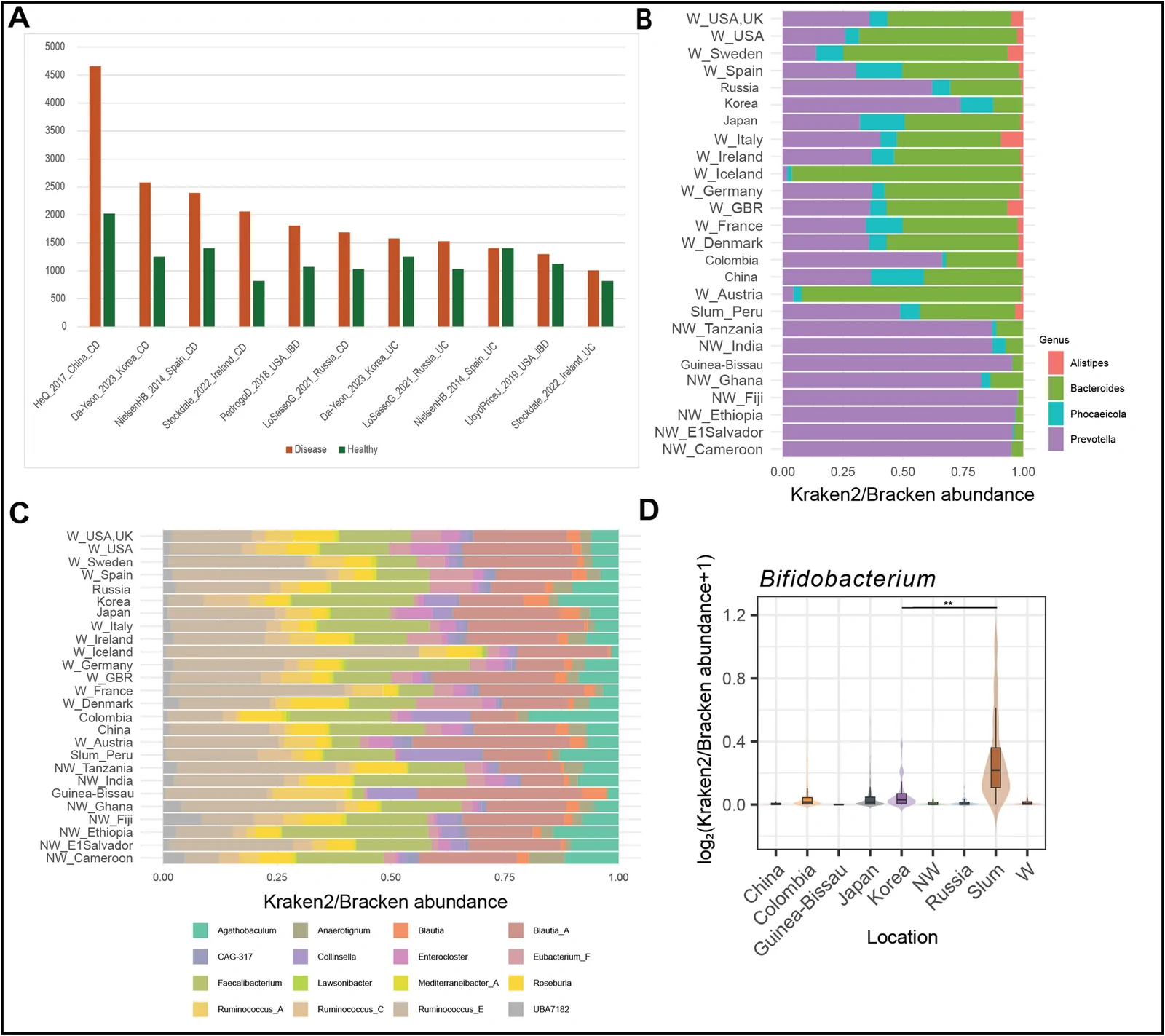
**(A)** Comparison of mean ARG abundance between healthy and IBD cohorts, including ulcerative colitis (UC) and Crohn’s disease (CD) subgroups across multiple datasets. **(B)** Relative abundance of dominant gut bacterial genera (*Bacteroides*, *Alistipes*, *Prevotella*, and *Phocaeicola*) across different global populations. **(C)** Relative abundance of bacterial genera contributing to the gut resistome among different population groups. **(D)** Relative abundance of *Bifidobacterium* across populations, showing its higher abundance in Peruvian slum cohort

To understand the differences in the gut resistome between healthy and disease groups, we analyzed mean ARG abundance across 52 case-control datasets. Among these, 40 datasets showed higher mean ARG abundance in disease cases, while 12 showed higher abundance in controls (Supplementary Table 5). Notably, inflammatory bowel disease (IBD) cohorts consistently showed higher mean ARG abundance in cases compared to healthy controls across studies (Fig. 6A, Supplementary Table 5). Furthermore, within IBD subtypes, Crohn’s disease (CD) patients exhibited higher ARG abundance compared to ulcerative colitis (UC) patients within the same study across populations, including China, USA, Spain, Russia, and Korea.

Differential abundance analysis using LEfSe (LDA score > 2, *p* < 0.05) further identified several ARGs consistently enriched in IBD compared to healthy samples across multiple datasets (Supplementary Table 5). These included genes associated with multidrug resistance and membrane-associated functions, such as *acrB*,* TolC*,* emrA*,* cpxA*,* mdtM*, and *CRP*. These genes showed higher relative abundance in CD and IBD samples across multiple studies (HeQ_China, USA_IBD_LloydP, Spain_CD, Russia_CD, and Da_Yeon_2023_Korea_CD) (Supplementary Table 5). Additionally, regulatory genes such as *H-NS* and *emrR* also showed higher relative abundance in IBD patients. These findings are consistent with previous reports indicating higher ARG abundance and diversity of ARGs in the gut microbiome of IBD patients compared to healthy individuals [55].

Overall, the higher relative abundance of ARGs observed in IBD may be associated with altered gut microbiome composition and the enrichment of opportunistic pathogens in IBD patients. Additionally, antibiotic treatment during IBD management may contribute to the expansion and persistence of ARGs in these populations.

### Abundance of ARG-harboring gut bacterial genomes varies across populations

To further understand the drivers of population-level resistome differences observed at the ARG level, we analyzed the distribution and relative abundance of 857 ARG-harboring UHGG microbial taxa across populations.

The widespread occurrence of tetracycline resistance genes across populations is consistent with their presence in dominant commensal taxa. For example, *tet(Q)* was predominantly harbored by Bacteroidota members, including *Prevotella hominis* and *Prevotella jejuni* Supplementary Fig. 4A, 4B), enriched in non-Western populations and *Bacteroides* and *Phocaeicola* species, prevalent in Western populations (Fig. 6B). Similarly, *tet(O)* homologs were widespread in Clostridia, including the *Blautia* and *Enterocloster* genera, which were prevalent across populations (Fig. 6C). The population-wide distribution of *tet(W)* might be associated with the presence of its homologs across various genomes, including *Collinsella*,* Agathobaculum*,* Ruminococcus*,* Massilioclostridium*,* Lawsonibacter*, *Faecalibacterium prausnitzii*, and Roseburia hominis, which were prevalent across multiple populations (Fig. 6C). These observations suggest that the broad distribution of tetracycline resistance genes reflects their presence in abundant commensal taxa. Homologs of *Bifidobacterium adolescentis rpoB* mutants were detected in 20 *Bifidobacterium* species. Consistent with this observation, the overall abundance of the *Bifidobacterium* genus was higher in Peruvian slum compared to other populations (Fig. 6D).

In contrast, macrolide resistance genes showed population-specific patterns. Genes such as *ErmB*, *ErmG*, and *Mef(En2)* were observed in taxa enriched in Western populations. For example, *ErmB* homologs were present in *Faecalibacterium prausnitzii*_*D*,* UBA3402 sp003478355*, and *KLE1615 sp900066985*, likely contributing to its higher abundance in Western populations (Supplementary Fig. 4C-E).

Similarly, the higher abundance of *Bacteroides thetaiotaomicron* and of *Bacteroides* and *Parabacteroides* (Fig. 6B) in Western cohorts may be associated with the higher abundance of *ErmG* and *Mef(En2)*, respectively. Enrichment of *Bacteroides uniformis* in Western compared to the non-Western populations may contribute to the higher relative abundance of *CblA-1* β-lactamase (Supplementary Fig. 4F).

*Butyricimonas paravirosa*, in which homologs of *OXA-347* were identified, showed higher abundance in Western than in non-Western populations (Supplementary Fig. 4G). Likewise, several Bacilli, Clostridia, and Bacteroidia members, including *AM07 − 15 sp003477405*,* Bacteroides sp90236237*,* Roseburia hominis*,* and UMGS966 sp900547185* that carry *APH(3’)−IIIa* homologs, also showed higher abundance in Western compared to non-Western group (Supplementary Fig. 4H-K).

Among non-Western populations, the higher abundance of *Prevotella* species, including *Prevotella copri_A*,* Prevotella hominis*, and *Prevotella sp900557255* is consistent with the observed enrichment of β-lactam resistance genes such as *CfxA6* (Supplementary Fig. 4A, 4 L, 4 M).

A higher relative abundance of *Escherichia and Salmonella* in Guinea-Bissau and Colombia was consistent with the higher relative abundance of *emrB* (multidrug efflux pump component) in these populations (Supplementary Fig. 5). Notably, these regions showed a higher relative abundance of Enterobacteriaceae members, including *Citrobacter*, *Escherichia*, *Klebsiella*, and *Salmonella*, which harbor homologs for multiple clinically relevant ARGs (Supplementary Fig. 5) in comparison to other populations.

These taxa carry genes associated with multidrug efflux systems (e.g., *mdt*,* acr*,* emr* families), membrane-associated resistance mechanisms (e.g., *eptA*,* msbA*,* gadX*,* leuO*,* LptD*), and additional clinically relevant resistance determinants such as *bacA* (bacitracin resistance) and multiple *Klebsiella pneumoniae* efflux-related genes (*OmpK37*,* KpnG*,* KpnE*,* KpnF*,* EC − 18*). The higher relative abundance of these Enterobacteriaceae members is thus consistent with the enrichment of multidrug resistance signatures observed in these populations.

### ARG-MGE co-occurrence suggests potential for horizontal gene transfer

While some gut bacteria are intrinsically resistant to antibiotics, others may acquire ARGs through HGT. Thus, to explore potential association between ARGs and mobile genetic elements (MGEs), we examined the co-occurrence of ARGs with MGEs in bacterial genomes obtained from the UHGG catalog.

Our analysis identified multiple ARGs located in proximity to MGEs on the same contigs (Supplementary Table 7). For example, the *lnuC* gene was frequently observed near MGEs and was detected in taxa such as *Streptococcus lutetiensis*, *Veillonella spp*., and *Dialister spp*. suggesting that these bacteria may act as potential reservoirs of ARGs associated with MGEs. Similarly, MLS resistance genes, including *ErmB*,* ErmG*, and *Erm(52)*, were also found in proximity to MGEs, indicating their potential mobility.

Tetracycline resistance genes were frequently associated with MGEs across various taxa. For example, *tet(Q)* was observed near MGEs in multiple members of the Bacteroidota phylum, while *tet(40)* and *tet(O/32/O)* were found near MGEs in Firmicutes_A. The *tet(W)* gene was associated with MGEs across multiple phyla, including Firmicutes_A, Firmicutes_C, Firmicutes, Proteobacteria, and Actinobacteriota, suggesting potential for interphylum dissemination.

The *CfxA6* gene was detected near MGEs in *Prevotella copri_A* and *Prevotella hominis*, indicating a possible role of HGT in its spread. In contrast, the *CblA-1* β-lactamase gene detected in *Bacteroides uniformis* did not show proximity to MGEs, suggesting limited evidence for mobility in this context. The macrolide resistance gene *mef(En2)* was also observed near MGEs and was present in both commensal bacteria and opportunistic pathogens such as *Bacteroides fragilis_A* of the Bacteroidota phylum.

While the contig-level data hint at potential ARG mobility, definitive conclusions are limited by the fragmented nature of MAGs [56]. However, the observed contig-level associations suggest the potential mobility of ARGs within the gut microbiome [57].

## Discussion

The human microbiome serves as a diverse reservoir of ARGs, and understanding the distribution of ARGs in the human microbiome across global populations is crucial for evaluating the risk of resistance dissemination and its implications for treatment strategies. Here, we carried out a large-scale population-wide analysis of the human gut microbiome using 10,230 gut metagenomes and 4,744 gut and 452 oral genomes, providing comprehensive insights into the taxonomic and population-level distribution of ARGs. Analysis of gut microbial genomes enabled the identification of key bacterial taxa carrying diverse ARG repertoires and revealed ARGs shared between commensal and pathogenic bacteria. Pathogenic species from the genera *Pseudomonas*, *Citrobacter*, *Klebsiella*, *Enterobacter*, and *Escherichia* harbored the highest diversity of ARGs, including clinically important ARGs, suggesting their role as dominant reservoirs of ARGs in the gut, consistent with their frequent association with clinical infections [58].

Beyond pathogenic species, commensal gut bacteria were also found to harbor ARGs. For example, the presence of cfxA β-lactamase homologs in *Prevotella* members, including *P. hominis* and *P. copri*, which dominate non-Western gut microbiomes, suggests that the core microbiome in these populations possesses intrinsic resistance to class A β-lactam antibiotics. Although these homologs are largely found among members of the Bacteroidales order, variants such as *cfxA2* were detected in opportunistic pathogens like *Bacteroides fragilis*, supporting the possibility of ARG exchange between commensals and pathogens. Similarly, a high abundance of *Bacteroides uniformis* in Western populations may explain the prevalence of *CblA-1*, class A β-lactam resistance gene, and *Mef(En2)* (macrolide, lincosamide resistance) in these populations. These findings indicate that core commensal bacteria in both Western and non-Western gut microbiomes carry distinct ARGs as part of their core genomes. Such intrinsic resistance may have evolved under long-term selective pressures within microbial communities, potentially shaped by natural antimicrobial compounds and interspecies interactions [59, 60]. Notably, *Mef(En2)* was detected in various commensals within the Bacteroidota phylum as well as in opportunistic pathogens such as *Bacteroides fragilis_A*, indicating its potential mobility between commensal and pathogenic bacteria.

Tetracycline-resistant ribosomal protection proteins, such as *tet(W)* and *tet(Q)*, were predominantly identified in gut commensals, including *Bacteroides ovatus*,* Faecalibacterium prausnitzii*, *Parabacteroides distasonis*, and several *Phocaeicola* species. Many of these species were abundant in Western populations. The widespread presence of these genes in dominant commensals suggests that the gut microbiota serves as a reservoir of tetracycline resistance related genes.

*rpoB* mutants conferring resistance to rifampicin, were detected in several *Bifidobacterium* species, including *B. adolescentis*,* B. bifidum*, and *B. breve*, among others that were abundant in the Peruvian slum. The widespread presence of these mutations across multiple Bifidobacteriaceae members highlights the potential of commensals to serve as reservoirs of resistance-associated variants, contributing to the baseline resistome of certain populations. Since *Bifidobacterium* species are also used as probiotics [61], the presence of rifampicin resistance may facilitate their ability to persist in the gut under antibiotic exposure, while also underscoring their potential role as reservoirs of resistance determinants.

Analysis of the oral bacterial genomes revealed a distinct resistome profile compared to the gut microbiome, aligning with previous observations [8]. ARGs, including *ermB* and *ermX* were the most commonly identified macrolide resistance genes in *Streptococcus*, *Rothia*, and *Prevotella* species [62]. Notably, the detection of *patA* and *patB* (fluoroquinolone efflux pumps) in *Streptococcus* species extends current knowledge, since these genes have not been extensively studied in the oral niche [63, 64]. Their presence might be associated with fluoroquinolone resistance. However, these findings need further investigation to assess their role in the oral environment. *Rothia mucilaginosa*, a common commensal of the oral cavity, was found to harbor *ermX* (macrolide resistance) and *tet(W)* (tetracycline resistance), indicating that oral commensals may serve as common carriers of ARGs [65]. The presence of a large number of ARGs, including multidrug efflux systems (e.g., AcrAB-TolC, MexAB-OprM) and β-lactamases (e.g., *ampC*, *OXA-846*) in *Pseudomonas aeruginosa* and *Escherichia flexneri* provides hints for further research to understand their potential role in antibiotic resistance within the oral environment.

Beyond the oral cavity, several commensals are shared between the mouth and gut. This overlap suggests the existence of an oral-gut axis. For example, *Prevotella* species, such as *P. jejuni* and *P. salivae*, were present in both the gut and oral microbiomes. This overlap suggests that some species of *Prevotella* are common commensals in both sites and may contribute to the distribution of antibiotic resistance along the oral-gut axis [66]. Extending this observation, several other species were also found to carry identical ARGs in both environments. For example, *Streptococcus mitis*,* Streptococcus oralis*, and *Streptococcus mutans* carried identical ARGs in both niches, indicating a conserved resistome. Similarly, species such as *Bifidobacterium dentium*,* Bifidobacterium scardovii*,* Haemophilus_D parainfluenzae*,* Parascardovia denticolens*,* Porphyromonas gingivalis*,* Prevotella jejuni*,* Prevotella salivae*, *Pseudomonas aeruginosa*, and *Scardovia wiggsiae* were present in both niches with shared ARGs. Together, these findings indicate that several bacterial species harbor common ARGs in both niches, suggesting potential overlap between the oral and gut resistome [67].

It is important to note that for genome level analyses separate genome catalogs, were used. Thus, differences in catalog size and assembly strategies between the gut and oral genome resources may influence the observed ARG patterns. Future studies incorporating larger and geographically diverse oral metagenomic datasets will enable more comprehensive comparisons between oral and gut resistomes.

Our large-scale analysis of gut metagenomic samples across diverse populations provided critical insights into the variation in ARG profiles across different geographical regions, countries, and health conditions. Consistent with earlier studies, our study confirms that ARGs against tetracyclines, beta-lactams, and MLS antibiotics are the most prevalent in the human gut microbiome [68, 69]. This widespread prevalence may reflect both the persistence of ancient resistance determinants within microbial communities and ongoing selective pressures from antibiotic exposure.

Metagenomic analysis revealed clear population-level trends in the abundance of ARGs and microbial composition. The *Prevotella* rich, non-Western microbiome was characterized by a higher abundance of cephamycin resistance gene (*CfxA6*), whereas Western populations, with *a Bacteroides*-rich microbiome, showed a higher abundance of cephalosporin resistance gene [36]. These results reflect how dominant members within the gut microbiome influence the resistome landscape. This effect underscores the role of microbial community structure, shaped by diet and lifestyle, in modulating the resistome across global populations. These results indicate an association between gut microbiome composition and ARG profiles, reinforcing the idea that microbial composition influences antibiotic resistance patterns across populations [70].

The variation in the abundance of various ARGs across populations likely reflects both antibiotic consumption patterns and broader ecological and socioeconomic factors. Chinese cohorts exhibited the highest abundance of macrolide and β-lactam-resistance genes (e.g., *ermB*, *ermG*, *CblA-1*, *OXA-347*), consistent with reports of widespread use of macrolides and cephalosporins in Chinese hospitals and exposure through food sources [12, 71–73]. Similarly, higher abundance of multidrug resistance genes in populations such as Guinea-Bissau, Peruvian slum, and Colombia likely reflects differences in microbial community composition, driven by poor sanitation, limited wastewater treatment, and gaps in the implementation of national AMR action plans [42, 43, 74, 75]. A higher representation of ARGs associated with macrolide and β-lactam resistance in Western populations may reflect long-term clinical exposure and historical selection pressures associated with the widespread use of antibiotics such as cephalosporins and macrolides [76].

While our findings show ARG-MGE co-localization within assembled genomes, due to the fragmented nature of MAGs and metagenomic assemblies, genomic proximity does not necessarily confirm physical mobilization or HGT. Therefore, further validation studies are needed to better understand the mobility potential of these ARGs.

Since this study integrates gut metagenomic datasets from multiple independent studies with heterogeneous sequencing depths, detection of low-abundance or rare ARGs may remain limited in some datasets. Although sequencing depth normalization was performed to improve cross-study comparability, future studies using deeper and more uniformly sequenced metagenomic datasets may provide improved resolution for identifying rare ARGs and low-abundance resistome features.

## Conclusion

This study provides a comprehensive insight into the human resistome across global populations by integrating large-scale genomic and metagenomic datasets. We show that ARGs in the microbiome are shaped by a combination of natural, evolutionarily maintained resistance genes (e.g., *CfxA*, *tet*, *rpoB* mutations) and acquired ARGs. A key finding is the role of commensals as both reservoirs and potential donors of ARGs. While pathogens such as *Pseudomonas*, *Klebsiella*, and *Escherichia* remain major carriers of clinically concerning ARGs, abundant commensals like *Prevotella* and *Bacteroides*, in non-Western and Western populations, respectively, contribute to the baseline resistome. Our analysis also highlights population-level differences in ARG composition that reflect microbial community composition and antibiotic usage patterns. Notably, *Prevotella*-rich non-Western microbiomes were enriched in cephamycin resistance (CfxA), whereas *Bacteroides*-dominated Western microbiomes carried higher levels of cephalosporin resistance (CblA). These findings emphasise the possible role of diet and lifestyle, along with antibiotic exposure in ARG distribution across populations. In addition, the higher prevalence of ARGs in Chinese cohorts may be linked to extensive antibiotic usage in both clinical and agricultural settings. Finally, the detection of shared ARGs across oral and gut microbiomes points to a potential oral–gut axis of resistance transmission. Together, these findings demonstrate that the human resistome is not only a reflection of pathogenic threats but also of the ecological and evolutionary dynamics of commensals.

## Supplementary Information

Supplementary Material 1

Supplementary Material 2

Supplementary Material 3

Supplementary Material 4

Supplementary Material 5

Supplementary Material 6

Supplementary Material 7

Supplementary Material 8

Supplementary Material 9

Supplementary Material 10

Supplementary Material 11

Supplementary Material 12

Supplementary Material 13

## Data Availability

Details of the metagenomic datasets, including project IDs and study information, are provided in Supplementary Table 1.
