# Supplementary material for: Insights into the dynamics of antibiotic resistance genes in the human gut microbiome across populations
Source: Gut Pathog. 2026 Sep 15;18:76. doi: 10.1186/s13099-026-00860-2 (PMC13591791; doi:10.1186/s13099-026-00860-2)
Supplement: Supplementary file 1 [file 13099_2026_860_MOESM10_ESM.pdf]

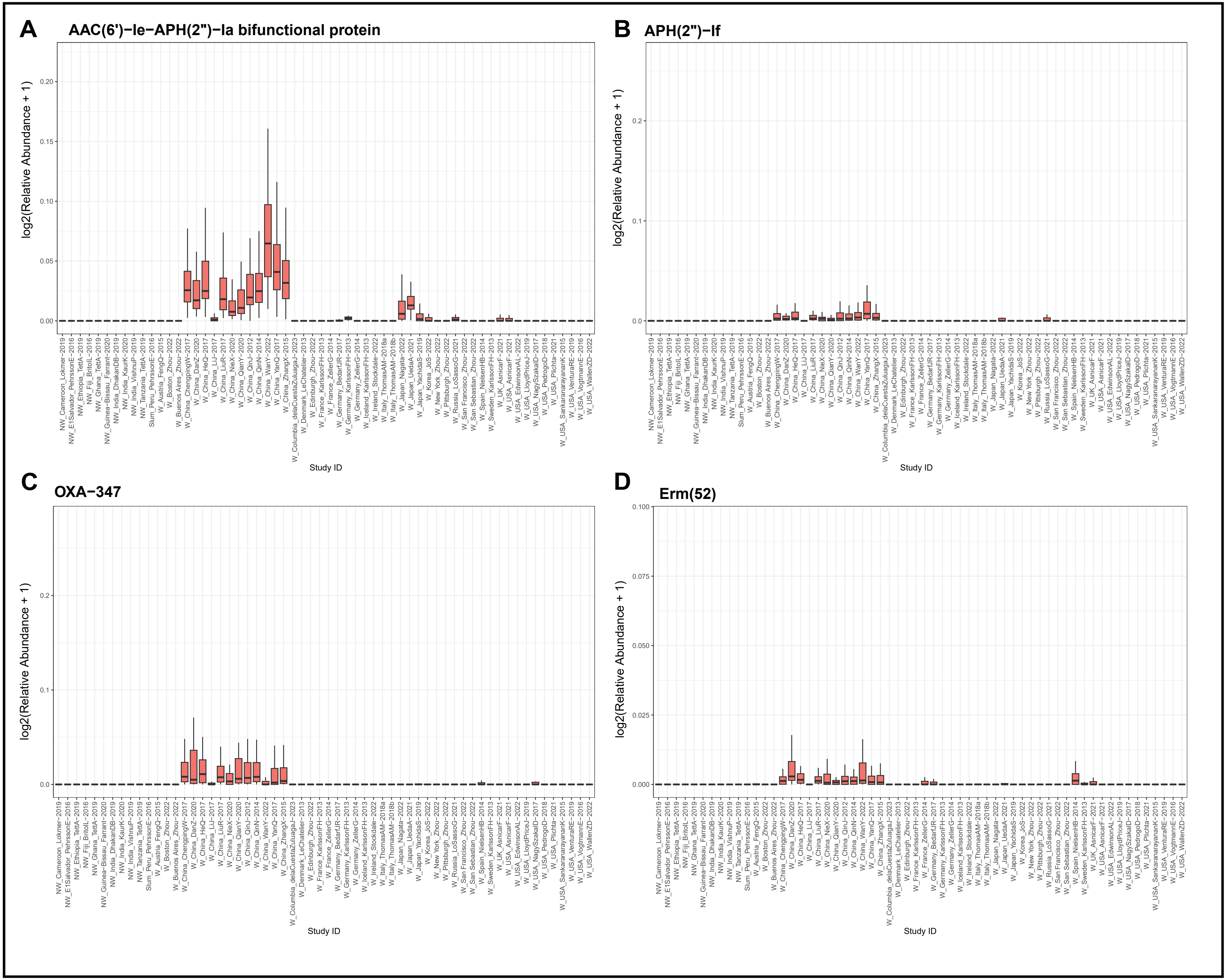
A
AAC(6')−Ie−APH(2")−Ia bifunctional protein
log2(Relative Abundance + 1)
0.20
0.15
0.10
0.05
0.00
Study ID
NW_Cameroon_Lokmer−2019
NW_E1Salvador_PehrssonE−2016
NW_Ethiopia_TettA−2019
NW_Fiji_BritoIL−2016
NW_Ghana_TettA−2019
NW_Guinea−Bissau_Farrant−2020
NW_India_DhakanDB−2019
NW_India_KaurK−2020
NW_India_VishnuP−2019
NW_Tanzania_TettA−2019
Slum_Peru_PehrssonE−2016
W_Austria_FengQ−2015
W_Boston_Zhou−2022
W_Buenos Aires_Zhou−2022
W_China_ChengpingW−2017
W_China_DanZ−2020
W_China_HeQ−2017
W_China_LiJ−2017
W_China_LiuR−2017
W_China_NieX−2020
W_China_QianY−2020
W_China_QinJ−2012
W_China_QinN−2014
W_China_WanY−2022
W_China_YanQ−2017
W_China_ZhangX−2015
W_Columbia_delaCuestaZuluagaJ−2023
W_Denmark_LeChatelier−2013
W_Edinburgh_Zhou−2022
W_France_KarlssonFH−2013
W_France_ZellerG−2014
W_Germany_BedarfJR−2017
W_Germany_KarlssonFH−2013
W_Germany_ZellerG−2014
W_Iceland_KarlssonFH−2013
W_Ireland_Stockdale−2022
W_Italy_ThomasAM−2018a
W_Italy_ThomasAM−2018b
W_Japan_Nagata−2022
W_Japan_UedaA−2021
W_Japan_YachidaS−2019
W_Korea_JoS−2022
W_New York_Zhou−2022
W_Pittsburgh_Zhou−2022
W_Russia_LoSassoG−2021
W_San Francisco_Zhou−2022
W_San Sebastian_Zhou−2022
W_Spain_NielsenHB−2014
W_Sweden_KarlssonFH−2013
W_UK_AsnicarF−2021
W_USA_AsnicarF−2021
W_USA_EdwinsonAL−2022
W_USA_LloydPriceJ−2019
W_USA_NagySzakalD−2017
W_USA_PedrogoD−2018
W_USA_Plichta−2021
W_USA_SankaranarayananK−2015
W_USA_VenturaRE−2019
W_USA_VogtmannE−2016
W_USA_WallenZD−2022
B
APH(2")−If
log2(Relative Abundance + 1)
0.2
0.1
0.0
Study ID
NW_Cameroon_Lokmer−2019
NW_E1Salvador_PehrssonE−2016
NW_Ethiopia_TettA−2019
NW_Fiji_BritoIL−2016
NW_Ghana_TettA−2019
NW_Guinea−Bissau_Farrant−2020
NW_India_DhakanDB−2019
NW_India_KaurK−2020
NW_India_VishnuP−2019
NW_Tanzania_TettA−2019
Slum_Peru_PehrssonE−2016
W_Austria_FengQ−2015
W_Boston_Zhou−2022
W_Buenos Aires_Zhou−2022
W_China_ChengpingW−2017
W_China_DanZ−2020
W_China_HeQ−2017
W_China_LiJ−2017
W_China_LiuR−2017
W_China_NieX−2020
W_China_QianY−2020
W_China_QinJ−2012
W_China_QinN−2014
W_China_WanY−2022
W_China_YanQ−2017
W_China_ZhangX−2015
W_Columbia_delaCuestaZuluagaJ−2023
W_Denmark_LeChatelier−2013
W_Edinburgh_Zhou−2022
W_France_KarlssonFH−2013
W_France_ZellerG−2014
W_Germany_BedarfJR−2017
W_Germany_KarlssonFH−2013
W_Germany_ZellerG−2014
W_Iceland_KarlssonFH−2013
W_Ireland_Stockdale−2022
W_Italy_ThomasAM−2018a
W_Italy_ThomasAM−2018b
W_Japan_Nagata−2022
W_Japan_UedaA−2021
W_Japan_YachidaS−2019
W_Korea_JoS−2022
W_New York_Zhou−2022
W_Pittsburgh_Zhou−2022
W_Russia_LoSassoG−2021
W_San Francisco_Zhou−2022
W_San Sebastian_Zhou−2022
W_Spain_NielsenHB−2014
W_Sweden_KarlssonFH−2013
W_UK_AsnicarF−2021
W_USA_AsnicarF−2021
W_USA_EdwinsonAL−2022
W_USA_LloydPriceJ−2019
W_USA_NagySzakalD−2017
W_USA_PedrogoD−2018
W_USA_Plichta−2021
W_USA_SankaranarayananK−2015
W_USA_VenturaRE−2019
W_USA_VogtmannE−2016
W_USA_WallenZD−2022
C
OXA−347
log2(Relative Abundance + 1)
0.2
0.1
0.0
Study ID
NW_Cameroon_Lokmer−2019
NW_E1Salvador_PehrssonE−2016
NW_Ethiopia_TettA−2019
NW_Fiji_BritoIL−2016
NW_Ghana_TettA−2019
NW_Guinea−Bissau_Farrant−2020
NW_India_DhakanDB−2019
NW_India_KaurK−2020
NW_India_VishnuP−2019
NW_Tanzania_TettA−2019
Slum_Peru_PehrssonE−2016
W_Austria_FengQ−2015
W_Boston_Zhou−2022
W_Buenos Aires_Zhou−2022
W_China_ChengpingW−2017
W_China_DanZ−2020
W_China_HeQ−2017
W_China_LiJ−2017
W_China_LiuR−2017
W_China_NieX−2020
W_China_QianY−2020
W_China_QinJ−2012
W_China_QinN−2014
W_China_WanY−2022
W_China_YanQ−2017
W_China_ZhangX−2015
W_Columbia_delaCuestaZuluagaJ−2023
W_Denmark_LeChatelier−2013
W_Edinburgh_Zhou−2022
W_France_KarlssonFH−2013
W_France_ZellerG−2014
W_Germany_BedarfJR−2017
W_Germany_KarlssonFH−2013
W_Germany_ZellerG−2014
W_Iceland_KarlssonFH−2013
W_Ireland_Stockdale−2022
W_Italy_ThomasAM−2018a
W_Italy_ThomasAM−2018b
W_Japan_Nagata−2022
W_Japan_UedaA−2021
W_Japan_YachidaS−2019
W_Korea_JoS−2022
W_New York_Zhou−2022
W_Pittsburgh_Zhou−2022
W_Russia_LoSassoG−2021
W_San Francisco_Zhou−2022
W_San Sebastian_Zhou−2022
W_Spain_NielsenHB−2014
W_Sweden_KarlssonFH−2013
W_UK_AsnicarF−2021
W_USA_AsnicarF−2021
W_USA_EdwinsonAL−2022
W_USA_LloydPriceJ−2019
W_USA_NagySzakalD−2017
W_USA_PedrogoD−2018
W_USA_Plichta−2021
W_USA_SankaranarayananK−2015
W_USA_VenturaRE−2019
W_USA_VogtmannE−2016
W_USA_WallenZD−2022
D
Erm(52)
log2(Relative Abundance + 1)
0.100
0.075
0.050
0.025
0.000
Study ID
NW_Cameroon_Lokmer−2019
NW_E1Salvador_PehrssonE−2016
NW_Ethiopia_TettA−2019
NW_Fiji_BritoIL−2016
NW_Ghana_TettA−2019
NW_Guinea−Bissau_Farrant−2020
NW_India_DhakanDB−2019
NW_India_KaurK−2020
NW_India_VishnuP−2019
NW_Tanzania_TettA−2019
Slum_Peru_PehrssonE−2016
W_Austria_FengQ−2015
W_Boston_Zhou−2022
W_Buenos Aires_Zhou−2022
W_China_ChengpingW−2017
W_China_DanZ−2020
W_China_HeQ−2017
W_China_LiJ−2017
W_China_LiuR−2017
W_China_NieX−2020
W_China_QianY−2020
W_China_QinJ−2012
W_China_QinN−2014
W_China_WanY−2022
W_China_YanQ−2017
W_China_ZhangX−2015
W_Columbia_delaCuestaZuluagaJ−2023
W_Denmark_LeChatelier−2013
W_Edinburgh_Zhou−2022
W_France_KarlssonFH−2013
W_France_ZellerG−2014
W_Germany_BedarfJR−2017
W_Germany_KarlssonFH−2013
W_Germany_ZellerG−2014
W_Iceland_KarlssonFH−2013
W_Ireland_Stockdale−2022
W_Italy_ThomasAM−2018a
W_Italy_ThomasAM−2018b
W_Japan_Nagata−2022
W_Japan_UedaA−2021
W_Japan_YachidaS−2019
W_Korea_JoS−2022
W_New York_Zhou−2022
W_Pittsburgh_Zhou−2022
W_Russia_LoSassoG−2021
W_San Francisco_Zhou−2022
W_San Sebastian_Zhou−2022
W_Spain_NielsenHB−2014
W_Sweden_KarlssonFH−2013
W_UK_AsnicarF−2021
W_USA_AsnicarF−2021
W_USA_EdwinsonAL−2022
W_USA_LloydPriceJ−2019
W_USA_NagySzakalD−2017
W_USA_PedrogoD−2018
W_USA_Plichta−2021
W_USA_SankaranarayananK−2015
W_USA_VenturaRE−2019
W_USA_VogtmannE−2016
W_USA_WallenZD−2022
