# Supplementary material for: Insights into the dynamics of antibiotic resistance genes in the human gut microbiome across populations
Source: Gut Pathog. 2026 Sep 15;18:76. doi: 10.1186/s13099-026-00860-2 (PMC13591791; doi:10.1186/s13099-026-00860-2)
Supplement: Supplementary file 2 [file 13099_2026_860_MOESM11_ESM.pdf]

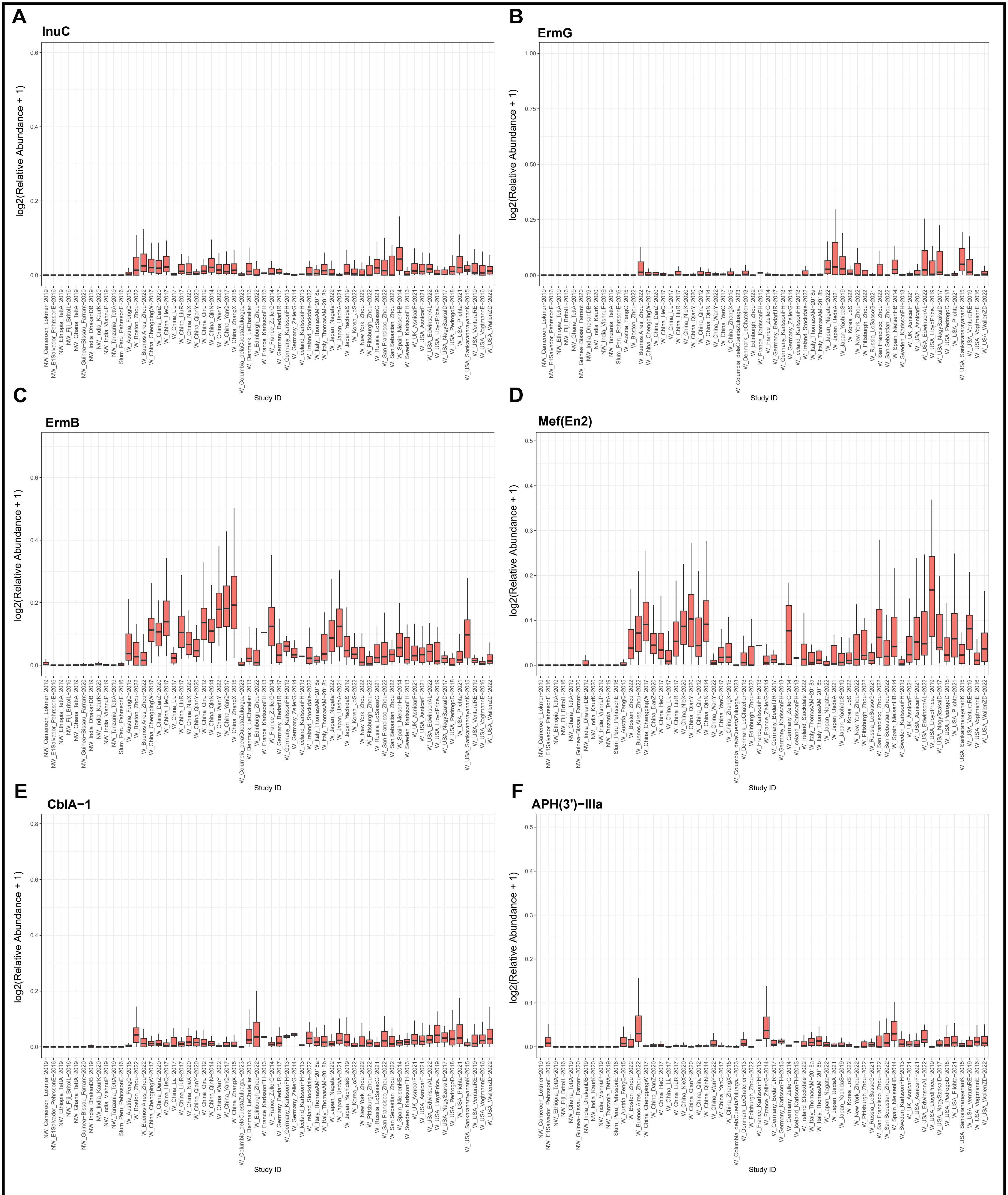

A
lnuC
log2(Relative Abundance + 1)
0.0
0.2
0.4
0.6
Study ID
B
ErmG
log2(Relative Abundance + 1)
0.00
0.25
0.50
0.75
1.00
Study ID
C
ErmB
log2(Relative Abundance + 1)
0.0
0.2
0.4
0.6
Study ID
D
Mef(En2)
log2(Relative Abundance + 1)
0.0
0.1
0.2
0.3
0.4
0.5
Study ID
E
CblA−1
log2(Relative Abundance + 1)
0.0
0.2
0.4
0.6
0.8
Study ID
F
APH(3')−IIIa
log2(Relative Abundance + 1)
0.0
0.1
0.2
0.3
0.4
0.5
Study ID
