# Supplementary material for: Insights into the dynamics of antibiotic resistance genes in the human gut microbiome across populations
Source: Gut Pathog. 2026 Sep 15;18:76. doi: 10.1186/s13099-026-00860-2 (PMC13591791; doi:10.1186/s13099-026-00860-2)
Supplement: Supplementary file 3 [file 13099_2026_860_MOESM12_ESM.pdf]

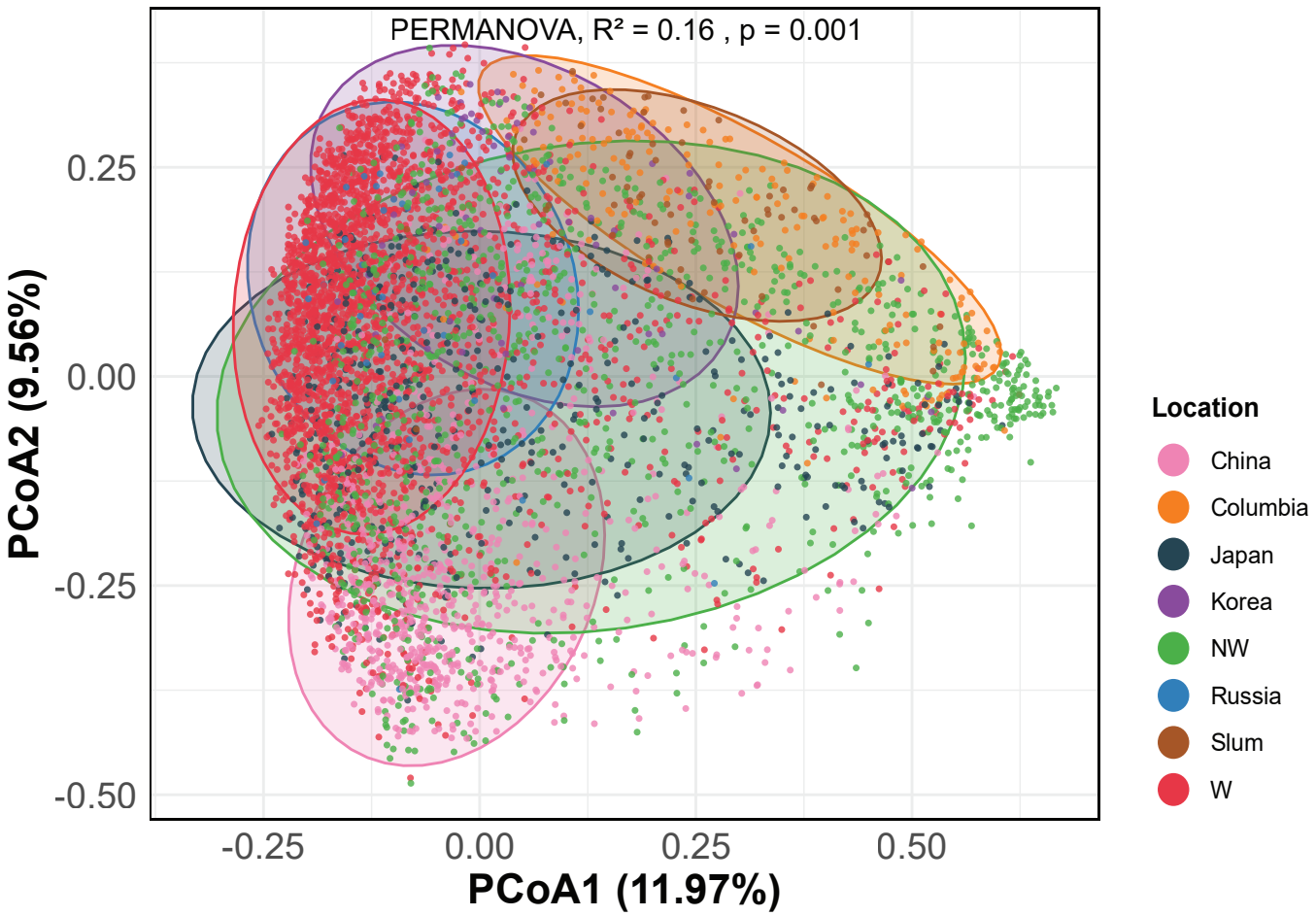

Mantel Test using qiime diversity beta-correlation	
Method	Spearman
Sample size	5388
Permutations	999
Alternative hypothesis	two-sided
Spearman rho	0.919357
p-value	0.001
