# Supplementary material for: Insights into the dynamics of antibiotic resistance genes in the human gut microbiome across populations
Source: Gut Pathog. 2026 Sep 15;18:76. doi: 10.1186/s13099-026-00860-2 (PMC13591791; doi:10.1186/s13099-026-00860-2)
Supplement: Supplementary file 4 [file 13099_2026_860_MOESM8_ESM.pdf]

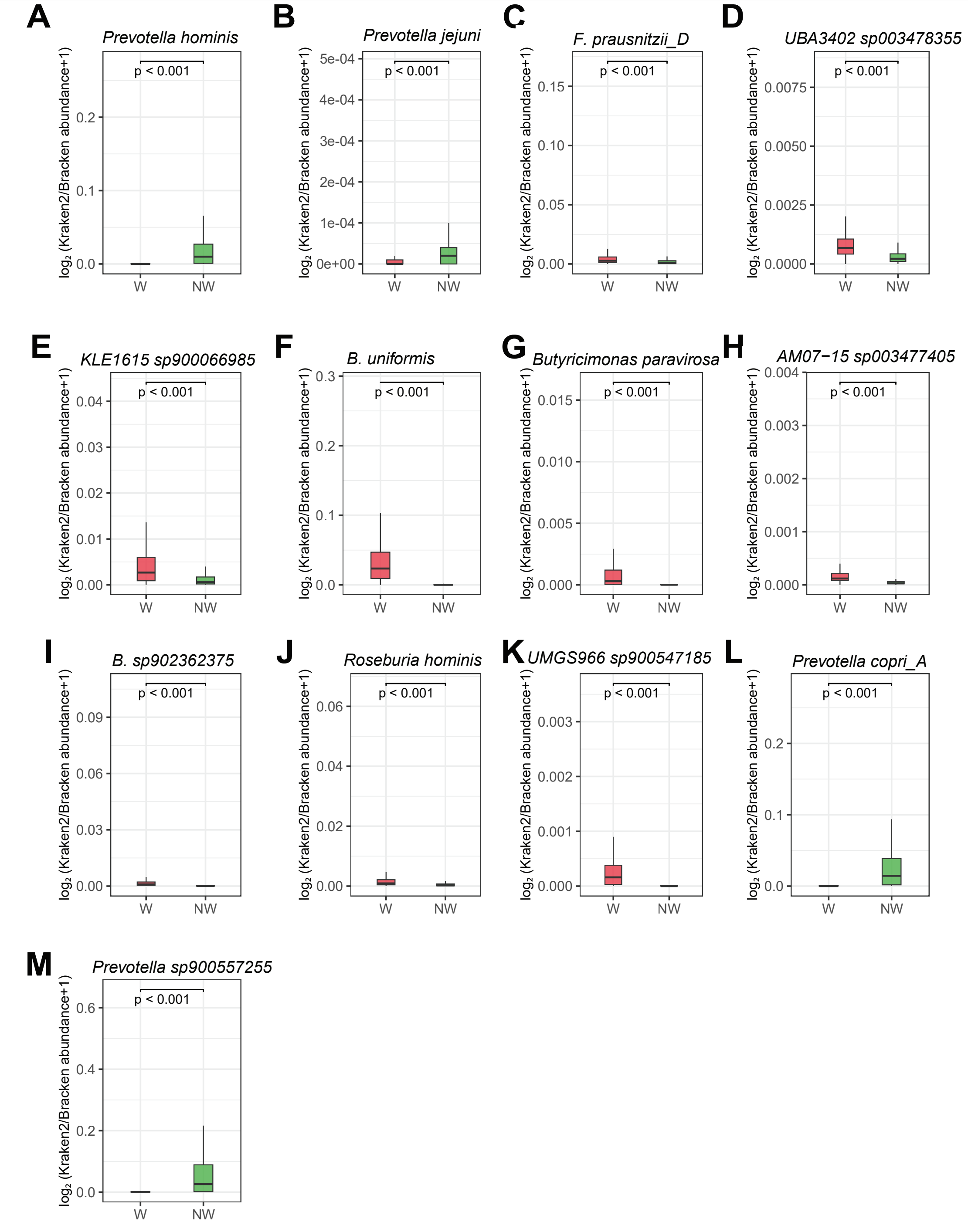
A
Prevotella hominis
B
Prevotella jejuni
C
F. prausnitzii_D
D
UBA3402 sp003478355
E
KLE1615 sp900066985
F
B. uniformis
G
Butyricimonas paravirosa
H
AM07−15 sp003477405
I
B. sp902362375
J
Roseburia hominis
K
UMGS966 sp900547185
L
Prevotella copri_A
M
Prevotella sp900557255
p < 0.001
log₂ (Kraken2/Bracken abundance+1)
W
NW
