# Supplementary material for: Insights into the dynamics of antibiotic resistance genes in the human gut microbiome across populations
Source: Gut Pathog. 2026 Sep 15;18:76. doi: 10.1186/s13099-026-00860-2 (PMC13591791; doi:10.1186/s13099-026-00860-2)
Supplement: Supplementary file 5 [file 13099_2026_860_MOESM9_ESM.pdf]

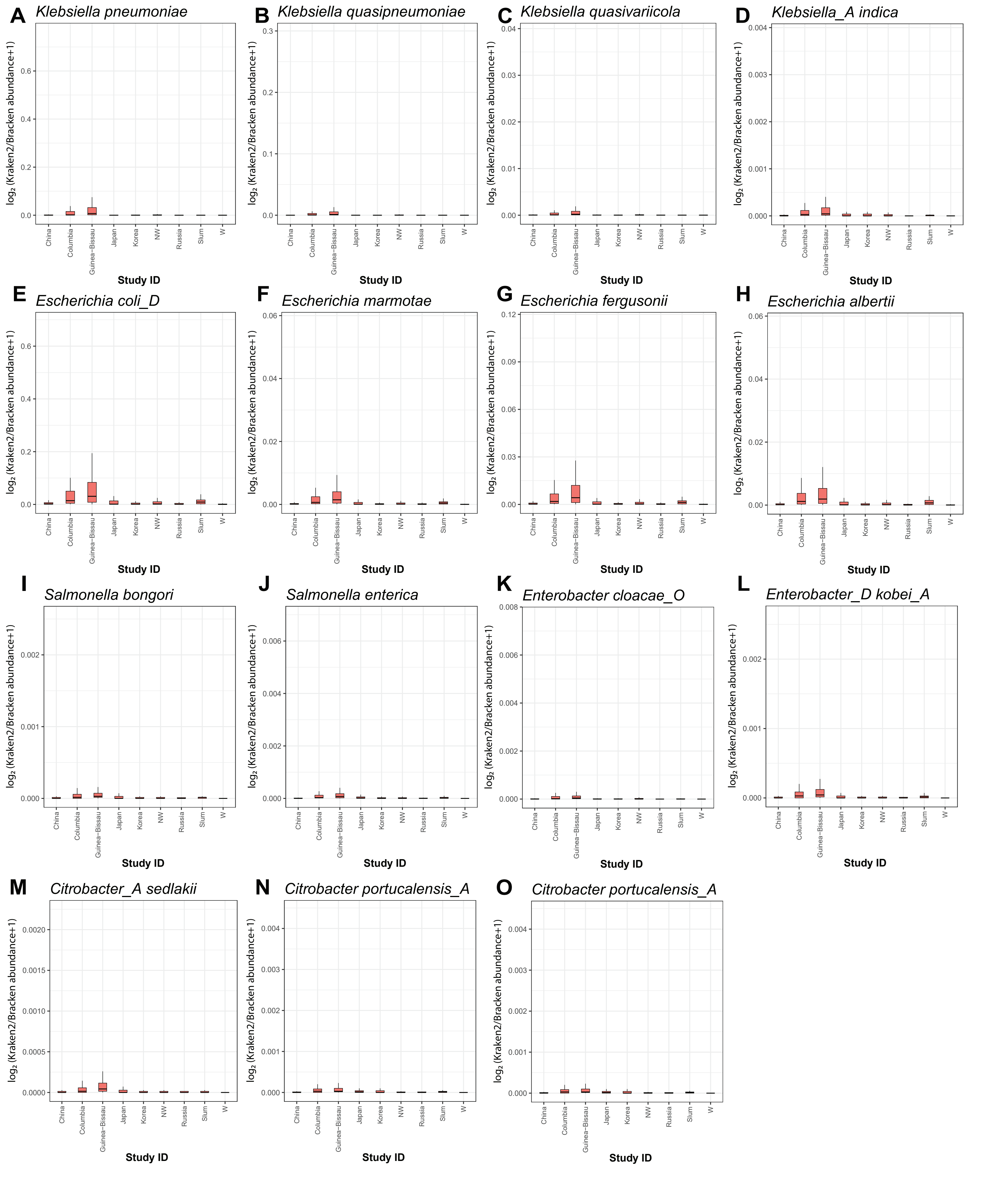

A Klebsiella pneumoniae
B Klebsiella quasipneumoniae
C Klebsiella quasivariicola
D Klebsiella_A indica
E Escherichia coli_D
F Escherichia marmotae
G Escherichia fergusonii
H Escherichia albertii
I Salmonella bongori
J Salmonella enterica
K Enterobacter cloacae_O
L Enterobacter_D kobei_A
M Citrobacter_A sedlakii
N Citrobacter portucalensis_A
O Citrobacter portucalensis_A
log2 (Kraken2/Bracken abundance+1)
Study ID
China
Columbia
Guinea−Bissau
Japan
Korea
NW
Russia
Slum
W
