# Supplementary material for: Insights into the dynamics of antibiotic resistance genes in the human gut microbiome across populations
Source: Gut Pathog. 2026 Sep 15;18:76. doi: 10.1186/s13099-026-00860-2 (PMC13591791; doi:10.1186/s13099-026-00860-2)
Supplement: Supplementary file 6 [file 13099_2026_860_MOESM13_ESM.docx]

**Supplementary Figure 1**. (PCoA) based on Bray-Curtis distances calculated using RPKM-based ARG abundance values showing the distribution of ARG profiles across China, Colombia, Japan, Korea, non-Western (NW), Russia, Slum, and Western (W) groups. Statistical significance was assessed using PERMANOVA (R² = 0.16, *p* = 0.001). A Mantel test using Spearman correlation (999 permutations) was performed to compare Bray-Curtis distance matrices generated from RPKM-based abundance and log₂-transformed relative abundance data, showing strong concordance between the two normalization approaches (Spearman ρ = 0.919, *p* = 0.001).

**Supplementary Figure 2**. **(A-F)** Boxplots showing a higher relative abundance of selected genes in studies from Western and Chinese populations compared to non-Western populations.

**Supplementary Figure 3**. **(A-D)** Boxplots showing the higher abundance of *AAC(6')-Ie-APH(2'')-Ia* bifunctional protein, *APH(2'')-If*, *OXA-347*, and *Erm(52)* in Chinese studies compared to other populations.

**Supplementary Figure 4**. **(A-M)** Boxplots showing the relative abundance of gut bacterial genomes across populations.

**Supplementary Figure 5**. Boxplots showing the higher relative abundance of various *Klebsiella* **(A-D)**, *Escherichia* **(E-H)**, *Salmonella* **(I,J)**, *Enterobacter* **(K,L)**, and *Citrobacter* **(M-O)** members in Colombia and Guinea-Bissau compared to other populations.

**Supplementary Table 1.** Summary of gut metagenomic datasets included in this study. The table provides the Project ID, disease category, disease abbreviation, number of healthy samples, number of disease samples, total samples, study ID, country of origin, and corresponding references for all 10,230 metagenomic samples analyzed.

**Supplementary Table 2.** Summary of gut bacterial species-representative genomes harbouring ARG homologs and their taxonomic distribution. The table includes information on species representatives harbouring ARGs along with their associated drug classes, resistance mechanisms, AMR gene families, and antibiotics.

**Supplementary Table 3.** Summary of ARG-containing UHGG genomes. The table provides a comprehensive overview of ARG distribution across UHGG gut genomes. It includes overall genome statistics; ARGs and the corresponding number of UHGG species representatives carrying each gene; ARG counts, drug class counts, and AMR family counts.

**Supplementary Table 4.** Summary of ARG-containing oral species-representative genomes. The table summarizes 64 human oral genomes (out of 452 species representatives) that possess ARGs, including associated drug classes, resistance mechanisms, AMR gene families, and antibiotics.

**Supplementary Table 5.** Summary of the top prevalent ARGs, PERMANOVA results, LEfSe outputs, and mean ARG abundances across studies. The file includes sheets listing the top 100 prevalent ARGs and their occurrence across samples, PERMANOVA results comparing population groups, mean ARG abundance across healthy and disease samples, and LEfSe results identifying differentially abundant ARGs among IBD, UC, CD, and healthy controls.

**Supplementary Table 6.** Differential abundance analysis and pairwise statistical comparisons of ARGs across populations. MaAsLin2 results identifying differentially abundant ARGs between population groups and pairwise Wilcoxon rank-sum test results with Benjamini-Hochberg adjusted p-values are provided.

**Supplementary Table7.** UHGG species-representative genomes with ARG and mobile genetic elements (MGEs). This table reports ARG homologs detected in each genome along with their corresponding drug classes, resistance mechanisms, genomic coordinates, alignment statistics, and annotations of nearby MGEs.
