# Supplementary material for: Insights into the dynamics of antibiotic resistance genes in the human gut microbiome across populations
Source: Gut Pathog. 2026 Sep 15;18:76. doi: 10.1186/s13099-026-00860-2 (PMC13591791; doi:10.1186/s13099-026-00860-2)
Supplement: Supplementary file 7 [file 13099_2026_860_MOESM1_ESM.docx]

**Supplementary Table 1**. Summary of human gut metagenomic datasets included in this study

| **Project_ID** | **Disease Category** | **Disease abbreviation** | **Healthy samples** | **Unhealthy samples** | **Total samples** | **Study ID** | **Country** | **References** |
| --- | --- | --- | --- | --- | --- | --- | --- | --- |
| PRJNA679346 | Alzheimer's Disease + Mild cognitive impairment | AD + MCI | 21 | 7 + 15 | 43 | UedaA-2021 | Japan | [1] |
| PRJNA375935 | Ankylosing spondylitis | AS | 114 | 94 | 208 | ChengpingW-2017 | China | [2] |
| PRJEB13870 | Hypertension |  | 41 | 98 | 139 | LiJ-2017 | China | [3] |
| PRJEB21612 | Hypertension |  | 56 | 60 | 116 | YanQ-2017 | China | [4] |
| PRJEB15371 | Crohn Disease | CD | 53 | 40 | 93 | HeQ-2017 | China | [5] |
| PRJNA813736 | Crohn Disease + Ulcerative Colitis | CD + UC | 48 | 19 + 20 | 87 | Stockdale-2022 | Ireland | [6] |
| PRJEB1220 | Crohn Disease + Ulcerative Colitis | CD + UC | 59 | 13 + 69 | 141 | NielsenHB-2014 | Spain | [7] |
| PRJNA893901 | Crohn Disease + Ulcerative Colitis | CD + UC | 42 | 30 + 32 | 104 | LoSassoG-2021 | Russia | [8] |
| PRJNA832909 | Pancreatic Ductal Adenocarcinoma | PDAC | 235 | 43 | 278 | Nagata-2022 | Japan | [9] |
| PRJEB6337 | Liver Cirrhosis | LC | 114 | 123 | 237 | QinN-2014 | China | [10] |
| PRJEB6997 | Rheumatoid Arthritis | RA | 91 | 90 | 181 | ZhangX-2015 | China | [11] |
| PRJNA598446 | Rheumatoid Arthritis | RA | 0 | 48 | 48 | GuptaVK-2020 | USA | [12] |
| PRJNA487636 | Inflammatory Bowel Disease + Rheumatoid Arthritis | IBD + RA | 64 | 64 + 25 | 153 | PedrogoD-2018 | USA | [13] |
| PRJNA398089 | Inflammatory Bowel Disease | IBD | 26 | 76 | 102 | LloydPriceJ-2019 | USA | [14] |
| PRJNA602729, PRJNA602731, PRJNA602732, PRJNA638403, PRJNA638404, PRJNA638405 | Graves' disease | GDI+GDII | 0 | 53+34 | 87 | ZhuQ-2021 | China | [15] |
| PRJNA743718 | Parkinson's Disease | PD | 74 | 82 | 156 | JoS-2022 | Korea | [16] |
| PRJNA433459 | Parkinson's Disease | PD | 40 | 37 | 77 | QianY-2020 | China | [17] |
| PRJNA834801 | Parkinson's Disease | PD | 234 | 491 | 725 | WallenZD-2022 | USA | [18] |
| PRJEB17784 | Parkinson's Disease | PD | 28 | 31 | 59 | BedarfJR-2017 | Germany | [19] |
| ERP115476 | Multiple Sclerosis | MS | 438 | 435 | 873 | Zhou-2022 | USA+ UK | [20] |
| PRJNA597839 | Obesity | OB | 30 | 32 | 62 | NieX-2020 | China | [21] |
| PRJEB12123 | Obesity | OB | 104 | 112 | 216 | LiuR-2017 | China | [22] |
| PRJEB58436 | Obesity +  Cardiometabolic Disease | OB+  CMD_NO | 203 | 206 | 409 | delaCuestaZuluagaJ-2023 | Colombia | [23,24] |
| PRJEB4336 | Obesity | OB | 123 | 169 | 292 | LeChatelier-2013 | Denmark | [25] |
| PRJNA705217 | Irritable Bowel Syndrome | IBS | 21 | 29 | 50 | EdwinsonAL-2022 | USA | [26] |
| PRJNA615162 | Immunoglobulin G4-related disease + Systemic Sclerosis | IgG4-RD + SSc | 165 | 58 + 90 | 313 | Plichta-2021 | USA | [27] |
| PRJNA299502 | Type 2 Diabetes | T2D | 17 | 19 | 36 | SankaranarayananK-2015 | USA | [28] |
| PRJNA422434 | Type 2 Diabetes | T2D | 70 | 70 | 140 | QinJ-2012 | China | [29] |
| PRJEB5224 | Type 1 and Type 2 Diabetes | T1D + T2D | 0 | 31 + 75 | 106 | LiJ-2014 | Denmark | [30] |
| PRJEB1786 | Type 2 Diabetes + Impaired Glucose Tolerance | T2D + IGT | 43 | 53+49 | 145 | KarlssonFH-2013 | Sweden+  Germany+  France+  Denmark | [31] |
| PRJNA379741 | Myalgic encephalomyelitis/Chronic Fatigue Syndrome | ME/CFS | 19 | 21 | 40 | NagySzakalD-2017 | USA | [32] |
| PRJEB14215 | Nonalcoholic fatty liver disease | NAFLD | 0 | 41 + 32 | 73 | HoylesL-2018 | Italy, Spain | [33] |
| PRJNA373901 | Nonalcoholic fatty liver disease | NAFLD | 0 | 85 | 85 | LoombaR-2017 | USA | [34] |
| ERP008729 | Colorectal Cancer + adenoma | CRC + adenoma | 63 | 46 + 47 | 156 | FengQ-2015 | Austria | [35] |
| NA | Autism spectrum disorder | ASD | 30 | 30 | 60 | DanZ-2020 | China | [36] |
| PRJNA447983 | Colorectal Cancer + adenoma | CRC + adenoma | 52 | 61+27 | 140 | ThomasAM-2018b,  ThomasAM-2018a | Italy | [37] |
| PRJEB12449 | Colorectal Cancer | CRC | 52 | 52 | 104 | VogtmannE-2016 | USA | [38] |
| PRJDB4176 | Colorectal Cancer | CRC | 246 | 185 | 431 | YachidaS-2019 | Japan | [39] |
| PRJNA531273 | Colorectal Cancer | CRC | 0 | 30 | 30 | GuptaA-2019 | India | [40] |
| PRJEB6070 | Colorectal Cancer | CRC | 93 | 90 | 183 | ZellerG-2014 | France + Germany | [41] |
| PRJNA531203 | Healthy Non-western |  | 31 | 0 | 31 | KaurK-2020 | India | [42] |
| PRJNA217052 | Healthy Non-western |  | 172 | 0 | 172 | BritoIL-2016 | Fiji | [43] |
| PRJNA529400,  PRJNA504891,  PRJNA529124 | Healthy Non-western |  | 68+  50+  44 | 0 | 162 | TettA-2019 | Tanzania+  Ethiopia+  Ghana | [44] |
| PRJNA300541 | Healthy Non-western |  | 112+  75 | 0+  0 | 187 | PehrssonE-2016 | El Salvador+  Peru | [45] |
| PRJEB27005 | Healthy Non-western |  | 57 | 0 | 57 | Lokmer-2019 | Cameroon | [46] |
| PRJEB39223 | Healthy western |  | 981 + 94 | 0 | 1075 | AsnicarF-2021 | UK + USA | [47] |
| PRJEB45799 | Healthy Non-western |  | 327 | 0 | 327 | Farrant-2020 | Guinea-Bissau | [48] |
| SRP100410 SRP100895  SRP119744  SRP120020 | Diarrhoea |  | 0 | 18 | 18 | KieserS_2018 | Bangladesh | [49] |
| PRJNA397112 | Healthy |  | 110 | 0 | 110 | DhakanDB-2019 | India | [50] |
| PRJNA547591 | Helminth infection |  | 0 | 89 | 89 | RubelMA_2020 | Cameroon | [51] |
| PRJNA715908, PRJNA397112 | Healthy Non-western |  | 70 | 0 | 70 | VishnuP-2019 | India | [52] |
| PRJNA686821 | Autism spectrum disorder | ASD | 64 | 64 | 128 | WanY-2022 | China | [53] |
| PRJNA438847 | Cystic fibrosis |  | 0 | 19 | 19 | Wang_2018 | Australia | [54] |
| PRJNA431482 | Behcet's disease |  | 45 | 24 | 69 | YeZ_2018 | China | [55] |
| PRJEB10878 | Colorectal cancer | CRC | 0 | 300 | 300 | Youwen-Qin-2024 | China | [56] |
| PRJNA486009 | Diarrhoea |  | 0 | 34 | 34 | SotoGiron_2021 | Ecuador | [57] |
| PRJNA945504, PRJKA220304 | Crohn Disease + Ulcerative Colitis | CD + UC | 50 | 167 + 234 | 451 | Da-Yeon_2023 | Korea | [58] |
| PRJEB28543 | Multiple Sclerosis | MS | 24 | 24 | 48 | VenturaRE-2019 | USA | [59] |

**Reference**

[1]	A. Ueda, S. Shinkai, H. Shiroma, Y. Taniguchi, S. Tsuchida, T. Kariya, T. Kawahara, Y. Kobayashi, N. Kohda, K. Ushida, A. Kitamura, T. Yamada, Identification of Faecalibacterium prausnitzii strains for gut microbiome-based intervention in Alzheimer’s-type dementia, Cell Reports Med. 2 (2021) 100398. https://doi.org/10.1016/j.xcrm.2021.100398.

[2]	C. Wen, Z. Zheng, T. Shao, L. Liu, Z. Xie, E. Le Chatelier, Z. He, W. Zhong, Y. Fan, L. Zhang, H. Li, C. Wu, C. Hu, Q. Xu, J. Zhou, S. Cai, D. Wang, Y. Huang, M. Breban, N. Qin, S.D. Ehrlich, Quantitative metagenomics reveals unique gut microbiome biomarkers in ankylosing spondylitis, Genome Biol. 18 (2017) 142. https://doi.org/10.1186/s13059-017-1271-6.

[3]	J. Li, F. Zhao, Y. Wang, J. Chen, J. Tao, G. Tian, S. Wu, W. Liu, Q. Cui, B. Geng, W. Zhang, R. Weldon, K. Auguste, L. Yang, X. Liu, L. Chen, X. Yang, B. Zhu, J. Cai, Gut microbiota dysbiosis contributes to the development of hypertension, Microbiome 5 (2017) 14. https://doi.org/10.1186/s40168-016-0222-x.

[4]	Q. Yan, Y. Gu, X. Li, W. Yang, L. Jia, C. Chen, X. Han, Y. Huang, L. Zhao, P. Li, Z. Fang, J. Zhou, X. Guan, Y. Ding, S. Wang, M. Khan, Y. Xin, S. Li, Y. Ma, Alterations of the Gut Microbiome in Hypertension, Front. Cell. Infect. Microbiol. 7 (2017). https://doi.org/10.3389/fcimb.2017.00381.

[5]	Q. He, Y. Gao, Z. Jie, X. Yu, J.M. Laursen, L. Xiao, Y. Li, L. Li, F. Zhang, Q. Feng, X. Li, J. Yu, C. Liu, P. Lan, T. Yan, X. Liu, X. Xu, H. Yang, J. Wang, L. Madsen, S. Brix, J. Wang, K. Kristiansen, H. Jia, Two distinct metacommunities characterize the gut microbiota in Crohn’s disease patients, Gigascience 6 (2017). https://doi.org/10.1093/gigascience/gix050.

[6]	S.R. Stockdale, R.S. Harrington, A.N. Shkoporov, E. V. Khokhlova, K.M. Daly, S.A. McDonnell, O. O’Reagan, J.A. Nolan, D. Sheehan, A. Lavelle, L.A. Draper, F. Shanahan, R.P. Ross, C. Hill, Metagenomic assembled plasmids of the human microbiome vary across disease cohorts, Sci. Rep. 12 (2022) 9212. https://doi.org/10.1038/s41598-022-13313-y.

[7]	H.B. Nielsen, M. Almeida, A.S. Juncker, S. Rasmussen, J. Li, S. Sunagawa, D.R. Plichta, L. Gautier, A.G. Pedersen, E. Le Chatelier, E. Pelletier, I. Bonde, T. Nielsen, C. Manichanh, M. Arumugam, J.-M. Batto, M.B. Quintanilha dos Santos, N. Blom, N. Borruel, K.S. Burgdorf, F. Boumezbeur, F. Casellas, J. Doré, P. Dworzynski, F. Guarner, T. Hansen, F. Hildebrand, R.S. Kaas, S. Kennedy, K. Kristiansen, J.R. Kultima, P. Léonard, F. Levenez, O. Lund, B. Moumen, D. Le Paslier, N. Pons, O. Pedersen, E. Prifti, J. Qin, J. Raes, S. Sørensen, J. Tap, S. Tims, D.W. Ussery, T. Yamada, P. Renault, T. Sicheritz-Ponten, P. Bork, J. Wang, S. Brunak, S.D. Ehrlich, Identification and assembly of genomes and genetic elements in complex metagenomic samples without using reference genomes, Nat. Biotechnol. 32 (2014) 822–828. https://doi.org/10.1038/nbt.2939.

[8]	G. Lo Sasso, L. Khachatryan, A. Kondylis, J.N.D. Battey, N. Sierro, N.A. Danilova, T. V Grigoryeva, M.I. Markelova, D.R. Khusnutdinova, A. V Laikov, I.I. Salafutdinov, Y.D. Romanova, M.N. Siniagina, I.Y. Vasiliev, E.A. Boulygina, V. V Solovyeva, E.E. Garanina, K. V Kitaeva, K.Y. Ivanov, D.S. Chulpanova, K.S. Kletenkov, A.R. Valeeva, A.K. Odintsova, M.D. Ardatskaya, R.A. Abdulkhakov, N. V Ivanov, M.C. Peitsch, J. Hoeng, S.R. Abdulkhakov, Inflammatory Bowel Disease–Associated Changes in the Gut: Focus on Kazan Patients, Inflamm. Bowel Dis. 27 (2021) 418–433. https://doi.org/10.1093/ibd/izaa188.

[9]	N. Nagata, S. Nishijima, Y. Kojima, Y. Hisada, K. Imbe, T. Miyoshi-Akiyama, W. Suda, M. Kimura, R. Aoki, K. Sekine, M. Ohsugi, K. Miki, T. Osawa, K. Ueki, S. Oka, M. Mizokami, E. Kartal, T.S.B. Schmidt, E. Molina-Montes, L. Estudillo, N. Malats, J. Trebicka, S. Kersting, M. Langheinrich, P. Bork, N. Uemura, T. Itoi, T. Kawai, Metagenomic Identification of Microbial Signatures Predicting Pancreatic Cancer From a Multinational Study, Gastroenterology 163 (2022) 222–238. https://doi.org/10.1053/j.gastro.2022.03.054.

[10]	N. Qin, F. Yang, A. Li, E. Prifti, Y. Chen, L. Shao, J. Guo, E. Le Chatelier, J. Yao, L. Wu, J. Zhou, S. Ni, L. Liu, N. Pons, J.M. Batto, S.P. Kennedy, P. Leonard, C. Yuan, W. Ding, Y. Chen, X. Hu, B. Zheng, G. Qian, W. Xu, S.D. Ehrlich, S. Zheng, L. Li, Alterations of the human gut microbiome in liver cirrhosis, Nature 513 (2014) 59–64. https://doi.org/10.1038/nature13568.

[11]	X. Zhang, D. Zhang, H. Jia, Q. Feng, D. Wang, D. Liang, X. Wu, J. Li, L. Tang, Y. Li, Z. Lan, B. Chen, Y. Li, H. Zhong, H. Xie, Z. Jie, W. Chen, S. Tang, X. Xu, X. Wang, X. Cai, S. Liu, Y. Xia, J. Li, X. Qiao, J.Y. Al-Aama, H. Chen, L. Wang, Q. Wu, F. Zhang, W. Zheng, Y. Li, M. Zhang, G. Luo, W. Xue, L. Xiao, J. Li, W. Chen, X. Xu, Y. Yin, H. Yang, J. Wang, K. Kristiansen, L. Liu, T. Li, Q. Huang, Y. Li, J. Wang, The oral and gut microbiomes are perturbed in rheumatoid arthritis and partly normalized after treatment, Nat. Med. 21 (2015) 895–905. https://doi.org/10.1038/nm.3914.

[12]	V.K. Gupta, M. Kim, U. Bakshi, K.Y. Cunningham, J.M. Davis, K.N. Lazaridis, H. Nelson, N. Chia, J. Sung, A predictive index for health status using species-level gut microbiome profiling, Nat. Commun. 11 (2020) 4635. https://doi.org/10.1038/s41467-020-18476-8.

[13]	D.A. Muñiz Pedrogo, J. Chen, B. Hillmann, P. Jeraldo, G. Al-Ghalith, V. Taneja, J.M. Davis, D. Knights, H. Nelson, W.A. Faubion, L. Raffals, P.C. Kashyap, An Increased Abundance of Clostridiaceae Characterizes Arthritis in Inflammatory Bowel Disease and Rheumatoid Arthritis: A Cross-sectional Study, Inflamm. Bowel Dis. 25 (2019) 902–913. https://doi.org/10.1093/ibd/izy318.

[14]	J. Lloyd-Price, C. Arze, A.N. Ananthakrishnan, M. Schirmer, J. Avila-Pacheco, T.W. Poon, E. Andrews, N.J. Ajami, K.S. Bonham, C.J. Brislawn, D. Casero, H. Courtney, A. Gonzalez, T.G. Graeber, A.B. Hall, K. Lake, C.J. Landers, H. Mallick, D.R. Plichta, M. Prasad, G. Rahnavard, J. Sauk, D. Shungin, Y. Vázquez-Baeza, R.A. White, J. Braun, L.A. Denson, J.K. Jansson, R. Knight, S. Kugathasan, D.P.B. McGovern, J.F. Petrosino, T.S. Stappenbeck, H.S. Winter, C.B. Clish, E.A. Franzosa, H. Vlamakis, R.J. Xavier, C. Huttenhower, Multi-omics of the gut microbial ecosystem in inflammatory bowel diseases, Nature 569 (2019) 655–662. https://doi.org/10.1038/s41586-019-1237-9.

[15]	Q. Zhu, Q. Hou, S. Huang, Q. Ou, D. Huo, Y. Vázquez-Baeza, C. Cen, V. Cantu, M. Estaki, H. Chang, P. Belda-Ferre, H.-C. Kim, K. Chen, R. Knight, J. Zhang, Compositional and genetic alterations in Graves’ disease gut microbiome reveal specific diagnostic biomarkers, ISME J. 15 (2021) 3399–3411. https://doi.org/10.1038/s41396-021-01016-7.

[16]	S. Jo, W. Kang, Y.S. Hwang, S.H. Lee, K.W. Park, M.S. Kim, H. Lee, H.J. Yoon, Y.K. Park, M. Chalita, J.H. Lee, H. Sung, J.-Y. Lee, J.-W. Bae, S.J. Chung, Oral and gut dysbiosis leads to functional alterations in Parkinson’s disease, Npj Park. Dis. 8 (2022) 87. https://doi.org/10.1038/s41531-022-00351-6.

[17]	Y. Qian, X. Yang, S. Xu, P. Huang, B. Li, J. Du, Y. He, B. Su, L.-M. Xu, L. Wang, R. Huang, S. Chen, Q. Xiao, Gut metagenomics-derived genes as potential biomarkers of Parkinson’s disease, Brain 143 (2020) 2474–2489. https://doi.org/10.1093/brain/awaa201.

[18]	Z.D. Wallen, A. Demirkan, G. Twa, G. Cohen, M.N. Dean, D.G. Standaert, T.R. Sampson, H. Payami, Metagenomics of Parkinson’s disease implicates the gut microbiome in multiple disease mechanisms, Nat. Commun. 13 (2022) 6958. https://doi.org/10.1038/s41467-022-34667-x.

[19]	J.R. Bedarf, F. Hildebrand, L.P. Coelho, S. Sunagawa, M. Bahram, F. Goeser, P. Bork, U. Wüllner, Functional implications of microbial and viral gut metagenome changes in early stage L-DOPA-naïve Parkinson’s disease patients, Genome Med. 2017 91 9 (2017) 1–13. https://doi.org/10.1186/S13073-017-0428-Y.

[20]	X. Zhou, R. Baumann, X. Gao, M. Mendoza, S. Singh, I. Katz Sand, Z. Xia, L.M. Cox, T. Chitnis, H. Yoon, L. Moles, S.J. Caillier, A. Santaniello, G. Ackermann, A. Harroud, R. Lincoln, R. Gomez, A. González Peña, E. Digga, D.J. Hakim, Y. Vazquez-Baeza, K. Soman, S. Warto, G. Humphrey, M. Farez, L.A. Gerdes, J.R. Oksenberg, S.S. Zamvil, S. Chandran, P. Connick, D. Otaegui, T. Castillo-Triviño, S.L. Hauser, J.M. Gelfand, H.L. Weiner, R. Hohlfeld, H. Wekerle, J. Graves, A. Bar-Or, B.A.C. Cree, J. Correale, R. Knight, S.E. Baranzini, Gut microbiome of multiple sclerosis patients and paired household healthy controls reveal associations with disease risk and course, Cell 185 (2022) 3467-3486.e16. https://doi.org/10.1016/j.cell.2022.08.021.

[21]	X. Nie, J. Chen, X. Ma, Y. Ni, Y. Shen, H. Yu, G. Panagiotou, Y. Bao, A metagenome-wide association study of gut microbiome and visceral fat accumulation, Comput. Struct. Biotechnol. J. 18 (2020) 2596–2609. https://doi.org/10.1016/j.csbj.2020.09.026.

[22]	R. Liu, J. Hong, X. Xu, Q. Feng, D. Zhang, Y. Gu, J. Shi, S. Zhao, W. Liu, X. Wang, H. Xia, Z. Liu, B. Cui, P. Liang, L. Xi, J. Jin, X. Ying, X. Wang, X. Zhao, W. Li, H. Jia, Z. Lan, F. Li, R. Wang, Y. Sun, M. Yang, Y. Shen, Z. Jie, J. Li, X. Chen, H. Zhong, H. Xie, Y. Zhang, W. Gu, X. Deng, B. Shen, X. Xu, H. Yang, G. Xu, Y. Bi, S. Lai, J. Wang, L. Qi, L. Madsen, J. Wang, G. Ning, K. Kristiansen, W. Wang, Gut microbiome and serum metabolome alterations in obesity and after weight-loss intervention, Nat. Med. 23 (2017) 859–868. https://doi.org/10.1038/nm.4358.

[23]	J. de la Cuesta-Zuluaga, V. Corrales-Agudelo, E.P. Velásquez-Mejía, J.A. Carmona, J.M. Abad, J.S. Escobar, Gut microbiota is associated with obesity and cardiometabolic disease in a population in the midst of Westernization, Sci. Rep. 8 (2018) 11356. https://doi.org/10.1038/s41598-018-29687-x.

[24]	J. de la Cuesta-Zuluaga, K.E. Huus, N.D. Youngblut, J.S. Escobar, R.E. Ley, Obesity is the main driver of altered gut microbiome functions in the metabolically unhealthy, Gut Microbes 15 (2023). https://doi.org/10.1080/19490976.2023.2246634.

[25]	E. Le Chatelier, T. Nielsen, J. Qin, E. Prifti, F. Hildebrand, G. Falony, M. Almeida, M. Arumugam, J.-M. Batto, S. Kennedy, P. Leonard, J. Li, K. Burgdorf, N. Grarup, T. Jørgensen, I. Brandslund, H.B. Nielsen, A.S. Juncker, M. Bertalan, F. Levenez, N. Pons, S. Rasmussen, S. Sunagawa, J. Tap, S. Tims, E.G. Zoetendal, S. Brunak, K. Clément, J. Doré, M. Kleerebezem, K. Kristiansen, P. Renault, T. Sicheritz-Ponten, W.M. de Vos, J.-D. Zucker, J. Raes, T. Hansen, E. Guedon, C. Delorme, S. Layec, G. Khaci, M. van de Guchte, G. Vandemeulebrouck, A. Jamet, R. Dervyn, N. Sanchez, E. Maguin, F. Haimet, Y. Winogradski, A. Cultrone, M. Leclerc, C. Juste, H. Blottière, E. Pelletier, D. LePaslier, F. Artiguenave, T. Bruls, J. Weissenbach, K. Turner, J. Parkhill, M. Antolin, C. Manichanh, F. Casellas, N. Boruel, E. Varela, A. Torrejon, F. Guarner, G. Denariaz, M. Derrien, J.E.T. van Hylckama Vlieg, P. Veiga, R. Oozeer, J. Knol, M. Rescigno, C. Brechot, C. M’Rini, A. Mérieux, T. Yamada, P. Bork, J. Wang, S.D. Ehrlich, O. Pedersen, Richness of human gut microbiome correlates with metabolic markers, Nature 500 (2013) 541–546. https://doi.org/10.1038/nature12506.

[26]	A.L. Edwinson, L. Yang, S. Peters, N. Hanning, P. Jeraldo, P. Jagtap, J.B. Simpson, T.-Y. Yang, P. Kumar, S. Mehta, A. Nair, M. Breen-Lyles, L. Chikkamenahalli, R.P. Graham, B. De Winter, R. Patel, S. Dasari, P. Kashyap, T. Griffin, J. Chen, G. Farrugia, M.R. Redinbo, M. Grover, Gut microbial β-glucuronidases regulate host luminal proteases and are depleted in irritable bowel syndrome, Nat. Microbiol. 7 (2022) 680–694. https://doi.org/10.1038/s41564-022-01103-1.

[27]	D.R. Plichta, J. Somani, M. Pichaud, Z.S. Wallace, A.D. Fernandes, C.A. Perugino, H. Lähdesmäki, J.H. Stone, H. Vlamakis, D.C. Chung, D. Khanna, S. Pillai, R.J. Xavier, Congruent microbiome signatures in fibrosis-prone autoimmune diseases: IgG4-related disease and systemic sclerosis, Genome Med. 13 (2021) 35. https://doi.org/10.1186/s13073-021-00853-7.

[28]	K. Sankaranarayanan, A.T. Ozga, C. Warinner, R.Y. Tito, A.J. Obregon-Tito, J. Xu, P.M. Gaffney, L.L. Jervis, D. Cox, L. Stephens, M. Foster, G. Tallbull, P. Spicer, C.M. Lewis, Gut Microbiome Diversity among Cheyenne and Arapaho Individuals from Western Oklahoma, Curr. Biol. 25 (2015) 3161–3169. https://doi.org/10.1016/j.cub.2015.10.060.

[29]	J. Wang, J. Qin, Y. Li, Z. Cai, S. Li, J. Zhu, F. Zhang, S. Liang, W. Zhang, Y. Guan, D. Shen, Y. Peng, D. Zhang, Z. Jie, W. Wu, Y. Qin, W. Xue, J. Li, L. Han, D. Lu, P. Wu, Y. Dai, X. Sun, Z. Li, A. Tang, S. Zhong, X. Li, W. Chen, R. Xu, M. Wang, Q. Feng, M. Gong, J. Yu, Y. Zhang, M. Zhang, T. Hansen, G. Sanchez, J. Raes, G. Falony, S. Okuda, M. Almeida, E. Lechatelier, P. Renault, N. Pons, J.M. Batto, Z. Zhang, H. Chen, R. Yang, W. Zheng, S. Li, H. Yang, S.D. Ehrlich, R. Nielsen, O. Pedersen, K. Kristiansen, J. Wang, A metagenome-wide association study of gut microbiota in type 2 diabetes, Nat. 2012 4907418 490 (2012) 55–60. https://doi.org/10.1038/nature11450.

[30]	J. Li, H. Jia, X. Cai, H. Zhong, Q. Feng, S. Sunagawa, M. Arumugam, J.R. Kultima, E. Prifti, T. Nielsen, A.S. Juncker, C. Manichanh, B. Chen, W. Zhang, F. Levenez, J. Wang, X. Xu, L. Xiao, S. Liang, D. Zhang, Z. Zhang, W. Chen, H. Zhao, J.Y. Al-Aama, S. Edris, H. Yang, J. Wang, T. Hansen, H.B. Nielsen, S. Brunak, K. Kristiansen, F. Guarner, O. Pedersen, J. Doré, S.D. Ehrlich, P. Bork, J. Wang, An integrated catalog of reference genes in the human gut microbiome, Nat. Biotechnol. 32 (2014) 834–841. https://doi.org/10.1038/nbt.2942.

[31]	F.H. Karlsson, V. Tremaroli, I. Nookaew, G. Bergström, C.J. Behre, B. Fagerberg, J. Nielsen, F. Bäckhed, Gut metagenome in European women with normal, impaired and diabetic glucose control, Nature 498 (2013) 99–103. https://doi.org/10.1038/nature12198.

[32]	D. Nagy-Szakal, B.L. Williams, N. Mishra, X. Che, B. Lee, L. Bateman, N.G. Klimas, A.L. Komaroff, S. Levine, J.G. Montoya, D.L. Peterson, D. Ramanan, K. Jain, M.L. Eddy, M. Hornig, W.I. Lipkin, Fecal metagenomic profiles in subgroups of patients with myalgic encephalomyelitis/chronic fatigue syndrome, Microbiome 5 (2017) 44. https://doi.org/10.1186/s40168-017-0261-y.

[33]	L. Hoyles, J.-M. Fernández-Real, M. Federici, M. Serino, J. Abbott, J. Charpentier, C. Heymes, J.L. Luque, E. Anthony, R.H. Barton, J. Chilloux, A. Myridakis, L. Martinez-Gili, J.M. Moreno-Navarrete, F. Benhamed, V. Azalbert, V. Blasco-Baque, J. Puig, G. Xifra, W. Ricart, C. Tomlinson, M. Woodbridge, M. Cardellini, F. Davato, I. Cardolini, O. Porzio, P. Gentileschi, F. Lopez, F. Foufelle, S.A. Butcher, E. Holmes, J.K. Nicholson, C. Postic, R. Burcelin, M.-E. Dumas, Molecular phenomics and metagenomics of hepatic steatosis in non-diabetic obese women, Nat. Med. 24 (2018) 1070–1080. https://doi.org/10.1038/s41591-018-0061-3.

[34]	R. Loomba, V. Seguritan, W. Li, T. Long, N. Klitgord, A. Bhatt, P.S. Dulai, C. Caussy, R. Bettencourt, S.K. Highlander, M.B. Jones, C.B. Sirlin, B. Schnabl, L. Brinkac, N. Schork, C.-H. Chen, D.A. Brenner, W. Biggs, S. Yooseph, J.C. Venter, K.E. Nelson, Gut Microbiome-Based Metagenomic Signature for Non-invasive Detection of Advanced Fibrosis in Human Nonalcoholic Fatty Liver Disease, Cell Metab. 25 (2017) 1054-1062.e5. https://doi.org/10.1016/j.cmet.2017.04.001.

[35]	Q. Feng, S. Liang, H. Jia, A. Stadlmayr, L. Tang, Z. Lan, D. Zhang, H. Xia, X. Xu, Z. Jie, L. Su, X. Li, X. Li, J. Li, L. Xiao, U. Huber-Schönauer, D. Niederseer, X. Xu, J.Y. Al-Aama, H. Yang, J. Wang, K. Kristiansen, M. Arumugam, H. Tilg, C. Datz, J. Wang, Gut microbiome development along the colorectal adenoma–carcinoma sequence, Nat. Commun. 2015 61 6 (2015) 1–13. https://doi.org/10.1038/ncomms7528.

[36]	Z. Dan, X. Mao, Q. Liu, M. Guo, Y. Zhuang, Z. Liu, K. Chen, J. Chen, R. Xu, J. Tang, L. Qin, B. Gu, K. Liu, C. Su, F. Zhang, Y. Xia, Z. Hu, X. Liu, Altered gut microbial profile is associated with abnormal metabolism activity of Autism Spectrum Disorder, Gut Microbes 11 (2020) 1246–1267. https://doi.org/10.1080/19490976.2020.1747329.

[37]	A.M. Thomas, P. Manghi, F. Asnicar, E. Pasolli, F. Armanini, M. Zolfo, F. Beghini, S. Manara, N. Karcher, C. Pozzi, S. Gandini, D. Serrano, S. Tarallo, A. Francavilla, G. Gallo, M. Trompetto, G. Ferrero, S. Mizutani, H. Shiroma, S. Shiba, T. Shibata, S. Yachida, T. Yamada, J. Wirbel, P. Schrotz-King, C.M. Ulrich, H. Brenner, M. Arumugam, P. Bork, G. Zeller, F. Cordero, E. Dias-Neto, J.C. Setubal, A. Tett, B. Pardini, M. Rescigno, L. Waldron, A. Naccarati, N. Segata, Metagenomic analysis of colorectal cancer datasets identifies cross-cohort microbial diagnostic signatures and a link with choline degradation, Nat. Med. 2019 254 25 (2019) 667–678. https://doi.org/10.1038/s41591-019-0405-7.

[38]	E. Vogtmann, X. Hua, G. Zeller, S. Sunagawa, A.Y. Voigt, R. Hercog, J.J. Goedert, J. Shi, P. Bork, R. Sinha, Colorectal Cancer and the Human Gut Microbiome: Reproducibility with Whole-Genome Shotgun Sequencing, PLoS One 11 (2016). https://doi.org/10.1371/JOURNAL.PONE.0155362.

[39]	S. Yachida, S. Mizutani, H. Shiroma, S. Shiba, T. Nakajima, T. Sakamoto, H. Watanabe, K. Masuda, Y. Nishimoto, M. Kubo, F. Hosoda, H. Rokutan, M. Matsumoto, H. Takamaru, M. Yamada, T. Matsuda, M. Iwasaki, T. Yamaji, T. Yachida, T. Soga, K. Kurokawa, A. Toyoda, Y. Ogura, T. Hayashi, M. Hatakeyama, H. Nakagama, Y. Saito, S. Fukuda, T. Shibata, T. Yamada, Metagenomic and metabolomic analyses reveal distinct stage-specific phenotypes of the gut microbiota in colorectal cancer, Nat. Med. 25 (2019) 968–976. https://doi.org/10.1038/S41591-019-0458-7.

[40]	A. Gupta, D.B. Dhakan, A. Maji, R. Saxena, V.P. P.K., S. Mahajan, J. Pulikkan, J. Kurian, A.M. Gomez, J. Scaria, K.R. Amato, A.K. Sharma, V.K. Sharma, Association of Flavonifractor plautii, a Flavonoid-Degrading Bacterium, with the Gut Microbiome of Colorectal Cancer Patients in India, MSystems 4 (2019). https://doi.org/10.1128/mSystems.00438-19.

[41]	G. Zeller, J. Tap, A.Y. Voigt, S. Sunagawa, J.R. Kultima, P.I. Costea, A. Amiot, J. Böhm, F. Brunetti, N. Habermann, R. Hercog, M. Koch, A. Luciani, D.R. Mende, M.A. Schneider, P. Schrotz‐King, C. Tournigand, J. Tran Van Nhieu, T. Yamada, J. Zimmermann, V. Benes, M. Kloor, C.M. Ulrich, M. von Knebel Doeberitz, I. Sobhani, P. Bork, Potential of fecal microbiota for early‐stage detection of colorectal cancer, Mol. Syst. Biol. 10 (2014). https://doi.org/10.15252/msb.20145645.

[42]	K. Kaur, I. Khatri, A. Akhtar, S. Subramanian, T.N.C. Ramya, Metagenomics analysis reveals features unique to Indian distal gut microbiota, PLoS One 15 (2020) e0231197. https://doi.org/10.1371/journal.pone.0231197.

[43]	I.L. Brito, S. Yilmaz, K. Huang, L. Xu, S.D. Jupiter, A.P. Jenkins, W. Naisilisili, M. Tamminen, C.S. Smillie, J.R. Wortman, B.W. Birren, R.J. Xavier, P.C. Blainey, A.K. Singh, D. Gevers, E.J. Alm, Mobile genes in the human microbiome are structured from global to individual scales, Nature 535 (2016) 435–439. https://doi.org/10.1038/nature18927.

[44]	A. Tett, K.D. Huang, F. Asnicar, H. Fehlner-Peach, E. Pasolli, N. Karcher, F. Armanini, P. Manghi, K. Bonham, M. Zolfo, F. De Filippis, C. Magnabosco, R. Bonneau, J. Lusingu, J. Amuasi, K. Reinhard, T. Rattei, F. Boulund, L. Engstrand, A. Zink, M.C. Collado, D.R. Littman, D. Eibach, D. Ercolini, O. Rota-Stabelli, C. Huttenhower, F. Maixner, N. Segata, The Prevotella copri Complex Comprises Four Distinct Clades Underrepresented in Westernized Populations, Cell Host Microbe 26 (2019) 666-679.e7. https://doi.org/10.1016/j.chom.2019.08.018.

[45]	E.C. Pehrsson, P. Tsukayama, S. Patel, M. Mejía-Bautista, G. Sosa-Soto, K.M. Navarrete, M. Calderon, L. Cabrera, W. Hoyos-Arango, M.T. Bertoli, D.E. Berg, R.H. Gilman, G. Dantas, Interconnected microbiomes and resistomes in low-income human habitats, Nature 533 (2016) 212–216. https://doi.org/10.1038/nature17672.

[46]	A. Lokmer, A. Cian, A. Froment, N. Gantois, E. Viscogliosi, M. Chabé, L. Ségurel, Use of shotgun metagenomics for the identification of protozoa in the gut microbiota of healthy individuals from worldwide populations with various industrialization levels, PLoS One 14 (2019) e0211139. https://doi.org/10.1371/journal.pone.0211139.

[47]	F. Asnicar, S.E. Berry, A.M. Valdes, L.H. Nguyen, G. Piccinno, D.A. Drew, E. Leeming, R. Gibson, C. Le Roy, H. Al Khatib, L. Francis, M. Mazidi, O. Mompeo, M. Valles-Colomer, A. Tett, F. Beghini, L. Dubois, D. Bazzani, A.M. Thomas, C. Mirzayi, A. Khleborodova, S. Oh, R. Hine, C. Bonnett, J. Capdevila, S. Danzanvilliers, F. Giordano, L. Geistlinger, L. Waldron, R. Davies, G. Hadjigeorgiou, J. Wolf, J.M. Ordovás, C. Gardner, P.W. Franks, A.T. Chan, C. Huttenhower, T.D. Spector, N. Segata, Microbiome connections with host metabolism and habitual diet from 1,098 deeply phenotyped individuals, Nat. Med. 27 (2021) 321. https://doi.org/10.1038/S41591-020-01183-8.

[48]	M. Valles-Colomer, A. Blanco-Míguez, P. Manghi, F. Asnicar, L. Dubois, D. Golzato, F. Armanini, F. Cumbo, K.D. Huang, S. Manara, G. Masetti, F. Pinto, E. Piperni, M. Punčochář, L. Ricci, M. Zolfo, O. Farrant, A. Goncalves, M. Selma-Royo, A.G. Binetti, J.E. Becerra, B. Han, J. Lusingu, J. Amuasi, L. Amoroso, A. Visconti, C.M. Steves, M. Falchi, M. Filosi, A. Tett, A. Last, Q. Xu, N. Qin, H. Qin, J. May, D. Eibach, M.V. Corrias, M. Ponzoni, E. Pasolli, T.D. Spector, E. Domenici, M.C. Collado, N. Segata, The person-to-person transmission landscape of the gut and oral microbiomes, Nature 614 (2023) 125–135. https://doi.org/10.1038/s41586-022-05620-1.

[49]	S. Kieser, S.A. Sarker, O. Sakwinska, F. Foata, S. Sultana, Z. Khan, S. Islam, N. Porta, S. Combremont, B. Betrisey, C. Fournier, A. Charpagne, P. Descombes, A. Mercenier, B. Berger, H. Brüssow, Bangladeshi children with acute diarrhoea show faecal microbiomes with increased Streptococcus abundance, irrespective of diarrhoea aetiology, Environ. Microbiol. 20 (2018) 2256–2269. https://doi.org/10.1111/1462-2920.14274.

[50]	D.B. Dhakan, A. Maji, A.K. Sharma, R. Saxena, J. Pulikkan, T. Grace, A. Gomez, J. Scaria, K.R. Amato, V.K. Sharma, The unique composition of Indian gut microbiome, gene catalogue, and associated fecal metabolome deciphered using multi-omics approaches, Gigascience 8 (2019). https://doi.org/10.1093/GIGASCIENCE/GIZ004.

[51]	M.A. Rubel, A. Abbas, L.J. Taylor, A. Connell, C. Tanes, K. Bittinger, V.N. Ndze, J.Y. Fonsah, E. Ngwang, A. Essiane, C. Fokunang, A.K. Njamnshi, F.D. Bushman, S.A. Tishkoff, Lifestyle and the presence of helminths is associated with gut microbiome composition in Cameroonians, Genome Biol. 2020 211 21 (2020) 1–32. https://doi.org/10.1186/S13059-020-02020-4.

[52]	V. Prasoodanan P. K, A.K. Sharma, S. Mahajan, D.B. Dhakan, A. Maji, J. Scaria, V.K. Sharma, Western and non-western gut microbiomes reveal new roles of Prevotella in carbohydrate metabolism and mouth–gut axis, Npj Biofilms Microbiomes 2021 71 7 (2021) 1–17. https://doi.org/10.1038/s41522-021-00248-x.

[53]	Y. Wan, T. Zuo, Z. Xu, F. Zhang, H. Zhan, D. Chan, T.F. Leung, Y.K. Yeoh, F.K.L. Chan, R. Chan, S.C. Ng, Underdevelopment of the gut microbiota and bacteria species as non-invasive markers of prediction in children with autism spectrum disorder, Gut 71 (2022) 910–918. https://doi.org/10.1136/GUTJNL-2020-324015.

[54]	Y. Wang, L.E.X. Leong, R.L. Keating, T. Kanno, G.C.J. Abell, F.M. Mobegi, J.M. Choo, S.L. Wesselingh, A.J. Mason, L.D. Burr, G.B. Rogers, Opportunistic bacteria confer the ability to ferment prebiotic starch in the adult cystic fibrosis gut, Gut Microbes 10 (2018) 367. https://doi.org/10.1080/19490976.2018.1534512.

[55]	Z. Ye, N. Zhang, C. Wu, X. Zhang, Q. Wang, X. Huang, L. Du, Q. Cao, J. Tang, C. Zhou, S. Hou, Y. He, Q. Xu, X. Xiong, A. Kijlstra, N. Qin, P. Yang, A metagenomic study of the gut microbiome in Behcet’s disease, Microbiome 2018 61 6 (2018) 1–13. https://doi.org/10.1186/S40168-018-0520-6.

[56]	Y. Qin, X. Tong, W.J. Mei, Y. Cheng, Y. Zou, K. Han, J. Yu, Z. Jie, T. Zhang, S. Zhu, X. Jin, J. Wang, H. Yang, X. Xu, H. Zhong, L. Xiao, P.R. Ding, Consistent signatures in the human gut microbiome of old- and young-onset colorectal cancer, Nat. Commun. 2024 151 15 (2024) 1–13. https://doi.org/10.1038/s41467-024-47523-x.

[57]	M.J. Soto-Girón, A. Peña-Gonzalez, J.K. Hatt, L. Montero, M. Páez, E. Ortega, S. Smith, W. Cevallos, G. Trueba, K.T. Konstantinidis, K. Levy, Gut Microbiome Changes with Acute Diarrheal Disease in Urban Versus Rural Settings in Northern Ecuador, Am. J. Trop. Med. Hyg. 104 (2021) 2275. https://doi.org/10.4269/AJTMH.20-0831.

[58]	D.Y. Kang, J.L. Park, M.K. Yeo, S.B. Kang, J.M. Kim, J.S. Kim, S.Y. Kim, Diagnosis of Crohn’s disease and ulcerative colitis using the microbiome, BMC Microbiol. 2023 231 23 (2023) 1–14. https://doi.org/10.1186/S12866-023-03084-5.

[59]	R.E. Ventura, T. Iizumi, T. Battaglia, M. Liu, G.I. Perez-Perez, J. Herbert, M.J. Blaser, Gut microbiome of treatment-naïve MS patients of different ethnicities early in disease course, Sci. Rep. 9 (2019) 16396. https://doi.org/10.1038/S41598-019-52894-Z.
